# Nanomaterials as Promising Additives for High-Performance 3D-Printed Concrete: A Critical Review

**DOI:** 10.3390/nano13091440

**Published:** 2023-04-22

**Authors:** Mehrdad Razzaghian Ghadikolaee, Elena Cerro-Prada, Zhu Pan, Asghar Habibnejad Korayem

**Affiliations:** 1 Nanomaterials Research Centre, School of Civil Engineering, Iran University of Science and Technology, Tehran 13114-16846, Iran; 2 Department of Electrical, Electronical, Automatic Control Engineering and Applied Physics, ETSIDI, Universidad Politécnica de Madrid, 28012 Madrid, Spain; 3 School of Civil and Transportation Engineering, Hebei University of Technology, Tianjin 300130, China; 4 Centre for Infrastructure Engineering, Western Sydney University, Penrith, NSW 2747, Australia

**Keywords:** 3D printing, digital fabrication, concrete, nanomaterials, additives

## Abstract

Three-dimensional (3D) printed concrete (3DPC), as one of the subset of digital fabrication, has provided a revolution in the construction industry. Accordingly, scientists, experts, and researchers in both academic and industry communities are trying to improve the performance of 3DPC. The mix design of all kinds of concrete has always been the most crucial property to reach the best efficiency. Recently, many studies have been performed to incorporate nano- and micro-scale additives to ameliorate the properties of 3DPC. The current study aims to present the main design properties of 3DPC and completely cover both fresh and hardened state characteristics of 3DPC containing different nano- and micro-additives. Our observations illustrate that nanomaterials can be mainly utilized as a thickener to ameliorate the thixotropic behavior and the structural build-up of 3DPC, resulting in higher yield stress and better viscosity recovery. Furthermore, each nanomaterial, through its unique impact, can provide lower porosity and permeability as well as better mechanical strengths for 3DPC. Although much research investigate the fresh properties of 3DPC containing nano and micro additives, future studies are needed to provide better insight into the impact of these kinds of additives on the hardened characteristics of 3DPC. In addition, researchers may devote more research to address the effects of the additives discussed herein on the performance of other kinds of 3DPC such as lightweight, self-compacting, etc. It should be noted that the effect mechanism of nanomaterials on the inter-layer bond strength of 3DPC is another crucial issue that should be investigated in future studies. Furthermore, nano-scale fillers from source of waste and biomass can be attractive additives for future research to achieve high performance of sustainable 3D-printed concrete.

## 1. Introduction

The construction industry is accountable for around 50% of solid waste [1], 38% of greenhouse gas emissions, and 12% of potable water use [2]. Among construction materials, cement has the most impact on climate change due to its responsibility for around 8% of total CO_2_ emissions [3]. According to the estimation for the future, the urban population will contain around 68% of the total residents of the world by 2050. Therefore, the footprint of the construction industry will increase over the next years, meaning more construction, more cementitious materials use [2], along with more CO_2_ emissions. Another critical concern is the unlimited natural resources that cause increase in the importance of reduction in natural materials consumption in the construction industry [4]. Hence, innovative sustainable materials, technologies, and strategies must be developed in the construction industry to overcome these challenges [2].

**Figure 1:**
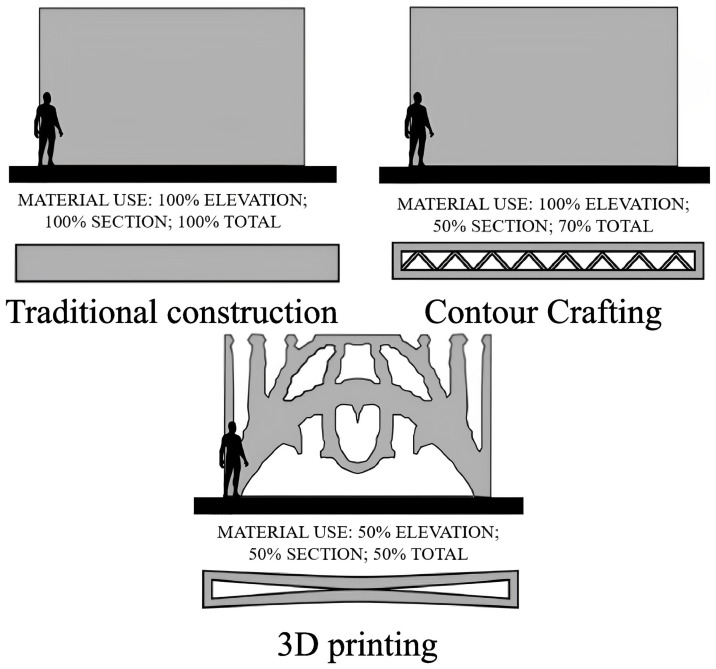
Difference between 3 types of construction. Reproduced with permission from [7].

Digital fabrication can be categorized as a promising solution to the sustainability mass-market requirements. Three-dimensional (3D) printing (3DP) has many advantages such as freedom in geometry, no formwork, less material usage, low waste, more eco-friendly and sustainable material selection, high rate of recycling, and cost-effectiveness. Moreover, 3DP is a one-step process to approach the demand for more sustainability and resource-efficiency in concrete construction practices. It provides ways to enhance the carbon footprints of buildings by reduction of their embodied and operating energy [5]. As can be seen in Figure 1, one of the most precious advantages of 3DP is that it can produce large-scale optimized structures without the employment of formworks, leading to reduced material usage, cost, and CO_2_ emissions [6,7].

**Figure 2:**
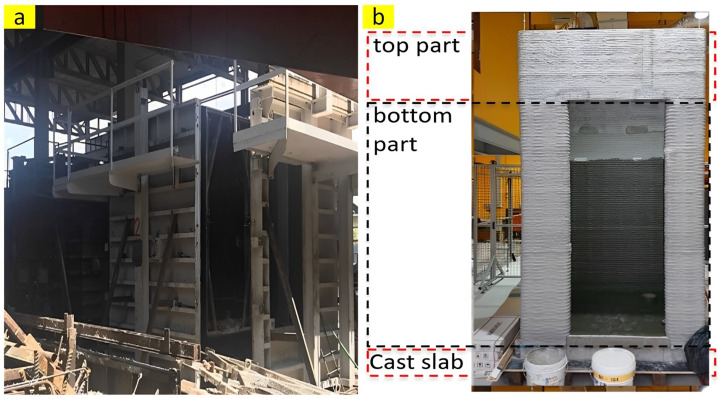
Metallic formwork for (**a**) precast bathroom and (**b**) 3D-printed bathroom. Adapted from [8].

In one comprehensive research, Yiwei Weng et al. [8] prepared a comparative study for manufacturing a cement-based bathroom unit fabricated by a precast approach and 3D printing. The results illustrate that a bathroom manufactured by 3D concrete printing (3DCP), as shown in Figure 2, achieves a reduction of approximately 86% in CO_2_ emission, 26% in overall cost, and 87% in energy consumption compared to that of the precast one. The bathroom fabricated by 3DCP also indicated lighter self-weight (26.2%↓) and higher productivity (48.1%↑) compared to that of the precast one.

Nowadays, nanotechnology has been developing, and nanomaterials have been mainly used in compounds with a number of conventional materials in order to ameliorate their characteristics. Many studies have been carried out on the impacts of nanomaterials on cementitious composites, most of which illustrate the improvement in mechanical properties, microstructure, and durability of cement-based materials [9,10,11,12,13,14,15,16]. Accordingly, these powerful materials have attracted the attention of many researchers across the world to make 3D printing technology closer to better characteristics. Recent studies illustrate that nanomaterials such as nano-silica [17], graphene [18], nanoclay [19], and nano-calcium-carbonate [20] can effectively ameliorate both fresh and hardened properties of 3D printable concrete (3DPC). These findings present the fact that nanomaterials can be named as a promising additive to achieve better properties of 3DPC.

The main objective of this study is to summarize recent research on the effect of nano and micro materials on the properties of 3D-printed concrete (3DPC). The first part of the paper is dedicated to describing the main fresh and hardened state characteristics, and mix proportion of 3DPC reported by previous studies. In the second part, we address the influence of nano- and micro-scale additives on the characteristics of 3DPC. Finally, the existing problems in current research and the future perspectives are presented.

## 2. Main Characteristics of 3D-Printed Concrete

### 2.1. Fresh State Properties

The fresh characteristics of 3DPC can be divided into three main stages in terms of printing, including before printing, during printing, and after printing [21]. Flow table, slump, setting time, and open time can be categorized as before-printing tests to analyze the printability of the mixture. The during-printing stage contains extrudability and buildability analysis. Finally, shape stability and green strength tests can be employed to evaluate the properties of 3DPC in the after-printing stage.

#### 2.1.1. Flowability

Flow table test, which can be carried out in accordance with ASTM-C230, is a well-known method to investigate the flowability of cementitious composites [22] and is also employed by researchers in order to verify the suitability of 3DPC for printing. As a guideline, the flow around 15.5±1 cm is suggested by previous studies as an optimal value for 3DPC, although it maybe needs to be changed for special cases. Some studies also performed slump and mini-slump experiments to analyze the workability and flowability of 3DPC. Chougan et al. [19] used a mini-slump cone apparatus with top and bottom diameters and height of 19, 38, and 57 mm, respectively. After one minute, they recorded the reduction of flow height as slump value.

#### 2.1.2. Setting Time, Open Time, and Hydration

**Figure 3:**
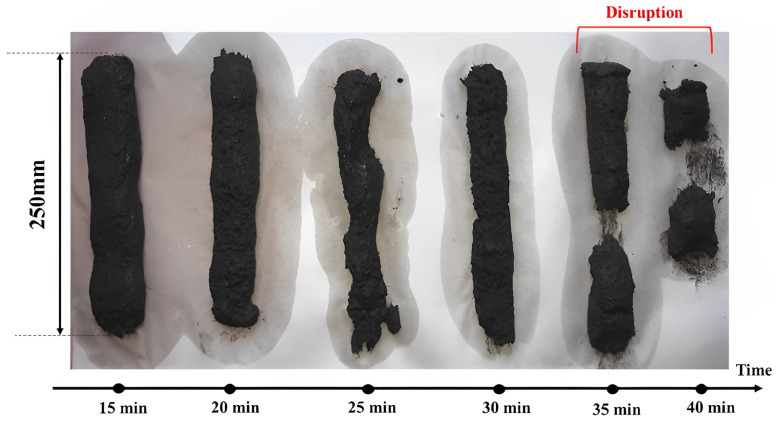
Open time test. Reproduced with permission from [18].

Setting and open time, such as flow table and slump, are other tests employed by researchers to evaluate the printability of the 3DPC mixture before starting the printing process [21]. The setting time of cementitious composites is a vital index used to determine printability time. This experiment can be performed with a Vicat needle following the procedure of ASTM-C191 for cement paste and a modified Vicat needle based on ASTM-C807 for cement mortar. Open time illustrates a period when a cement-based mixture can show proper workability for printing [18]. To approximately analyze this property, some researchers employed rheology, slump, or Vicat needle tests over specific interval times [23,24,25]. However, Chougan et al. [18] introduce a method to investigate the actual open time of 3DPC. According to Figure 3, they printed mixtures with a width and length of 2.4 and 25 cm, respectively, with resting time intervals of 5 min before observing a disruption. Furthermore, the hydration study of the binder phase of 3DPC can be assessed by some common methods such as isothermal conduction calorimeter, TAM air conductivity calorimeter, X-ray diffraction (XRD), thermogravimetric analysis (TGA), and differential thermal analysis (DTA).

#### 2.1.3. Extrudability

**Figure 4:**
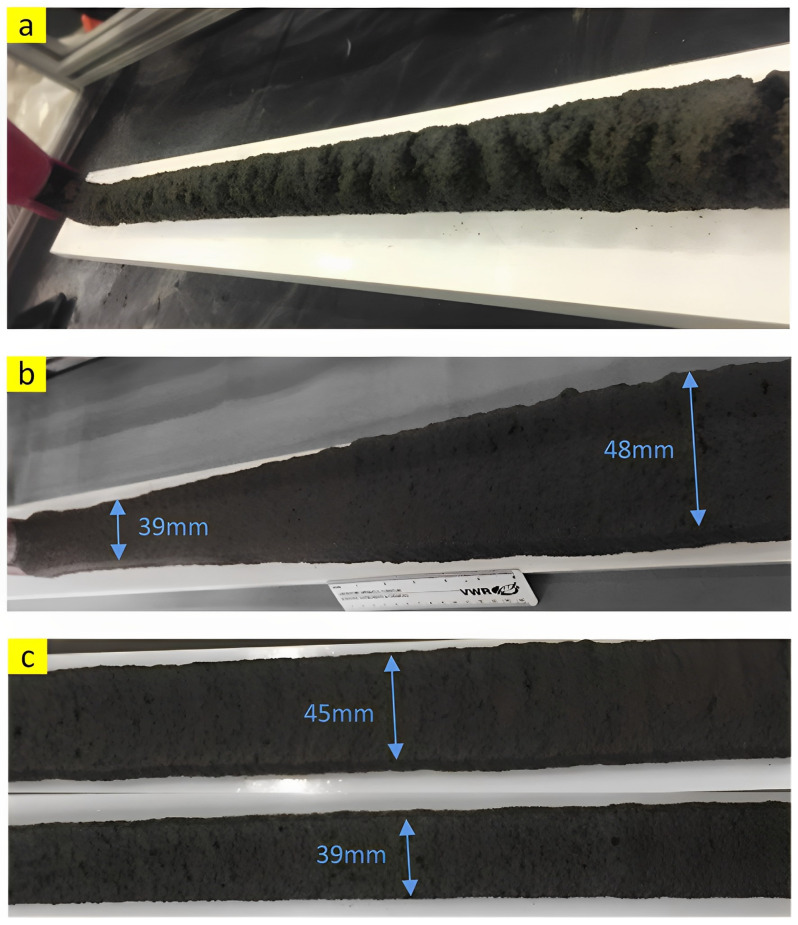
(**a**) Discontinuity (tearing), (**b**) dimension conformity, and (**c**) dimension consistency. Reproduced with permission from [26].

The capability of a concrete mixture to be continuously printed without any visible segregation when the mixture has been pumping through a nozzle can be named as extrudability. Although there are still no specific guidelines and standards to precisely measure this property, researchers employed some qualitative (visual) and quantitative methods to analyze the extrudability of 3DPC. Kazemian et al. [26] reported a visual procedure that checks the printed layer in terms of discontinuity (tearing), dimension conformity and consistency, and visible and squared edges (Figure 4). According to this method, a printed mixture could be considered acceptable if the three aforementioned requirements are passed. Some studies used quantitive procedures through measuring the needed extrusion pressure for starting extrusion [20,27]. Based on the method presented by Chu et al. [20], dead weight gradually increases on the top of the piston until the concrete starts to flow out through the nozzle of the tube. Finally, the extrusion pressure is calculated by dividing the maximum dead weight by the top surface of the piston.

#### 2.1.4. Buildability

**Figure 5:**
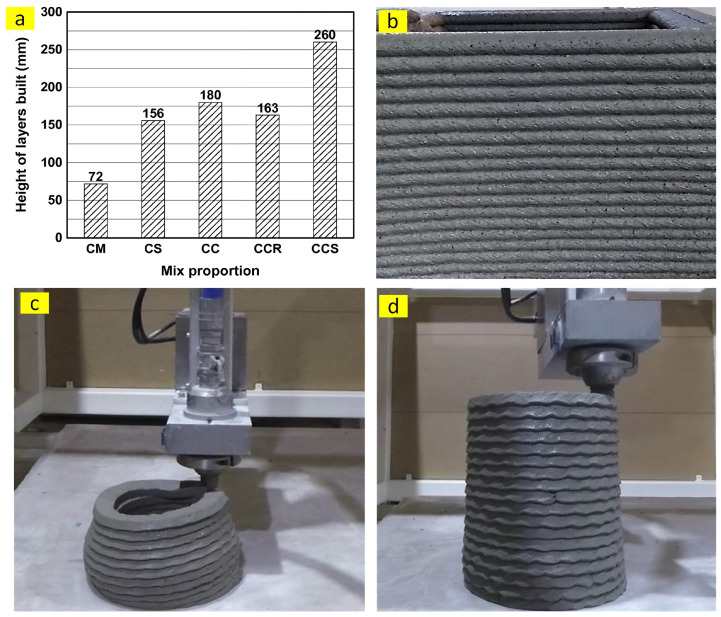
(**a**,**b**) Height of printed layers, reproduced with permission from [32], and (**c**,**d**) number of printed layers, reproduced with permission from [30].

Buildability refers to the appropriate early strength and stiffness of printed layers to simultaneously withstand four main forces, including its own weight, the weight of top layers, the pressure of the nozzle, and other external loads without any visible deformation and collapse [28]. The unsuitable buildability of 3DPC might be ascribed to three mechanisms, including elastic buckling, plastic collapse, and their combination [29]. The occurrence of buckling failure is caused by progressive lateral deformations [28] and the stiffness of the mixture, while the yield stress of fresh 3DPC affects the plastic collapse [29]. Hence, the elastic modulus of 3DPC is important to reach acceptable buildability in addition to the yield strength. The most common method to investigate the buildability of 3DPC, which is used by many studies [18,30,31,32,33], is to print the mixture layer by layer until visible deformation, failure, or collapse. In this situation, as is apparent in Figure 5, the maximum number [30] or the height [32] of printed layers is reported as the buildability index.

#### 2.1.5. Shape Retention

**Figure 6:**
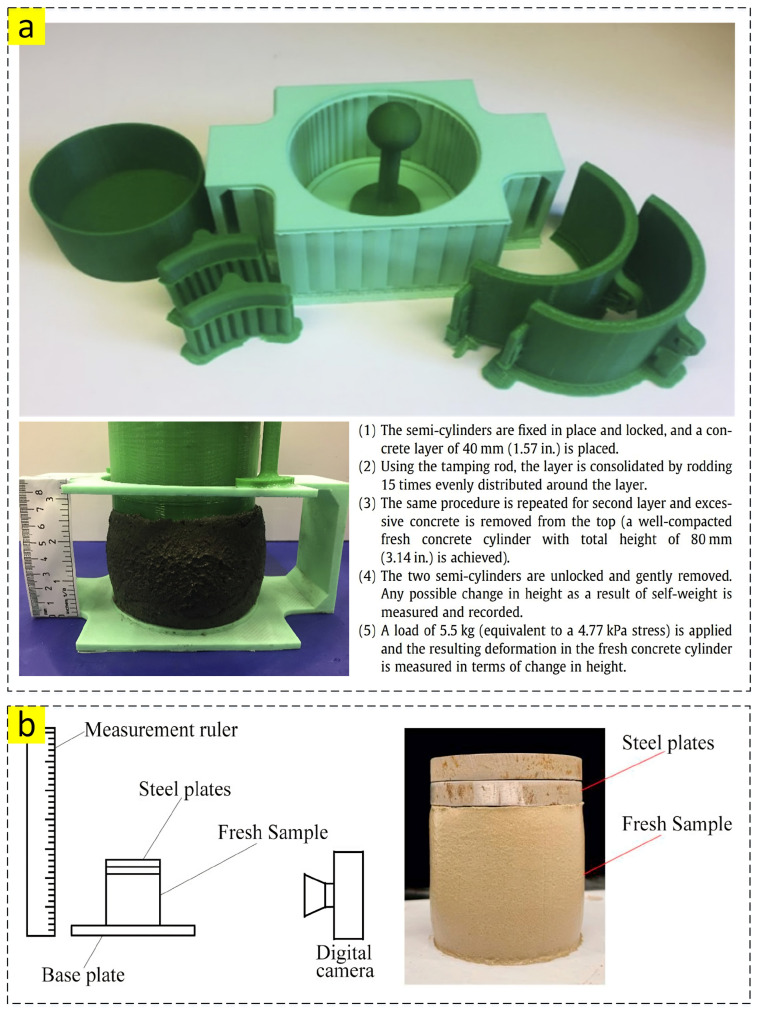
(**a**) Apparatus and stages of shape stability test [26]; (**b**) Second method of shape stability test. Reproduced with permission from [34].

Shape retention or the shape stability test refers to the ability of a fresh mixture to resist deformations when applying the the load of top-printed layers and extrusion pressure [26], and it is employed by several researchers to scrutinize the fresh properties of 3DPC. As illustrated in Figure 6a, Kazemian et al. developed a unique method named “Cylinder stability” for the shape stability test. They filled the specific mold with a mixture in two main layers and compacted each layer by rodding 15 times, and then slightly lifted the mold up. Finally, a load of 5.5 kg was applied on the top of the sample and the height drop was reported as shape stability. Another procedure (Figure 6b), which is more well-known among researchers [32,34,35,36], has some differences. In this method, a cylinder mold is slowly filled with fresh mixture, and then the mixture is compacted. After carefully lifting the mold up, a steel plate with a specific weight is applied on the top of the de-molded specimen to provide a deforming situation for the sample. After a specific time (around 30 s), to consider the gap of printing between layers, another steel plate is added to the top of the sample, and this process continues until the mixture collapses. It should be noted that the deformation of the sample is recorded using e.g., a digital camera in each stage of adding the steel plate [34]. The second method is almost like the green strength test explained in the following section.

#### 2.1.6. Green Strength

**Figure 7:**
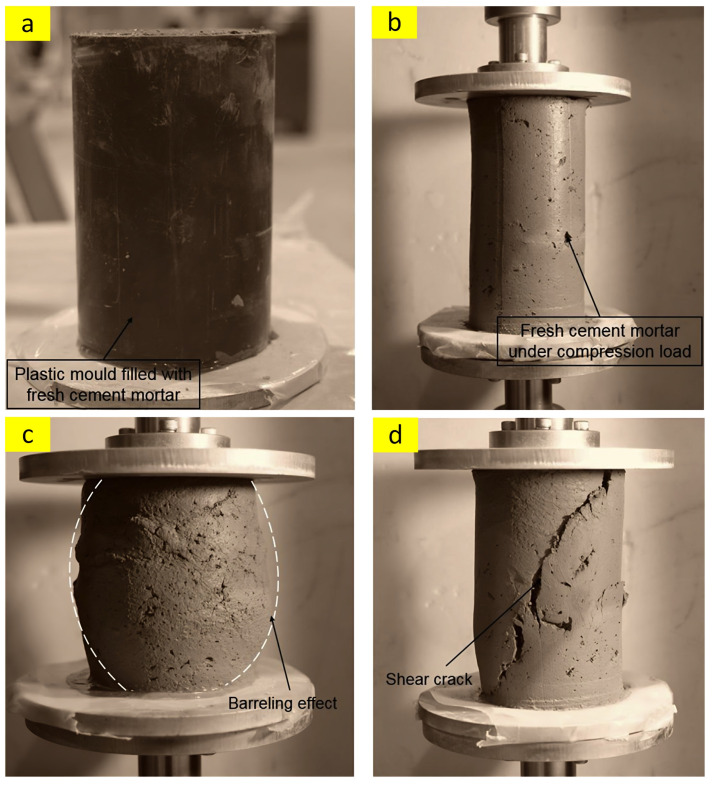
(**a**) Mold filled with fresh mixture, (**b**) fresh mixture under a compression load, (**c**,**d**) typical failure after resting time of 30 and 150 min, respectively. Reproduced with permission from [38].

Green strength can be carried out in order to evaluate the resistance of the fresh mixture against a load that is gradually applied. Zhang et al. [32] reported a simple procedure to estimate the green strength. This method consists of five stages including (1) filling a cylinder with a dimension of 10 × 10 cm with a fresh mixture, (2) compacting the molded concrete for approximately 30 s on a vibration stand, (3) de-molding the mixture and placing a cylinder barrel on top of the de-molded sample, (4) gradually adding sand until obvious deformation or collapse, and then (5) converting the weight of sand to green strength. It should be noted that the green strength of five separate specimens of the same batch at the intervals of 0, 15, 30, 45, and 60 min is calculated. In addition to that, recent studies [37,38,39] used the uniaxial unconfined compressive test to investigate the green strength of 3DPC (Figure 7).

#### 2.1.7. Rheology

**Figure 8:**
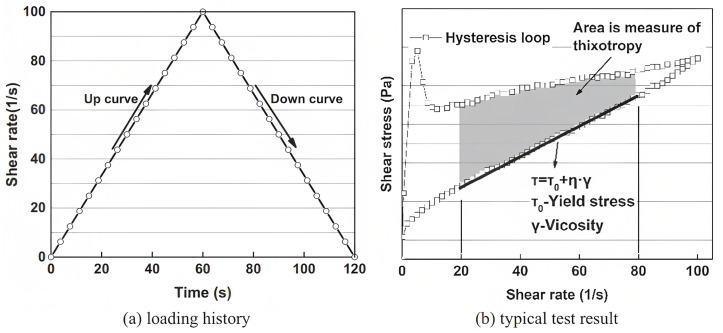
Hysteresis loop and typical result. Reproduced with permission from [32].

Rheology is one of the crucial tests for this kind of concrete because it can present three practical properties of 3CPC, including yield stress, viscosity, and thixotropy, that can effectively help researchers [32,40,41] to understand the fresh behavior of the mixture. The most common method employed by researchers to evaluate the rheological characteristics is one type of shear history to form a hysteresis loop using the up and down curve. To reach this purpose, a pre-shear is applied to the specimen, and then the shear rate increases from 0 to 100 s${}^{-1}$ within 60 s (up curve). Finally, the down curve can be created by decreasing the shear rate from 0 to 100 s${}^{-1}$ within the next 60 s. It should be noted that this is only a guideline and the aforementioned shear rate and time can be changed in each study. The Bingham model, a simple but practical equation (Equation (1)), can be utilized to reach the rheological properties of the mixture. Accordingly, viscosity γ and yield stress $\tau_{0}$ are estimated by fitting the down curve between the shear rate of 20 and 80. In addition to that, the shadow area (as shown in Figure 8) between the up and down curves is named thixotropy, which can be ascribed to the energy stored in the specimen’s flocs [32].

(1) $$\tau =\tau_{0}+\eta \cdot \gamma$$

**Figure 9:**
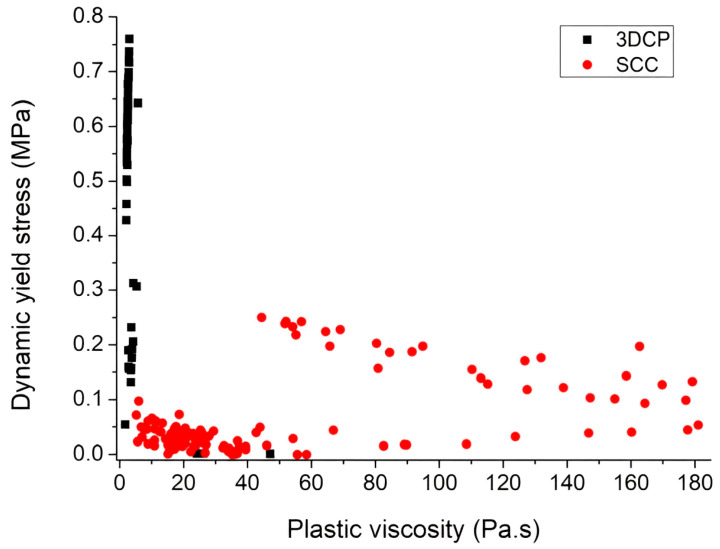
A comparison of rheological parameters between 3DPC and self-compacting concrete (SCC). Reproduced with permission from [42].

Table 1 and Table 2 present the range of static yield stress, dynamic yield stress, and plastic viscosity as rheological parameters for different 3DPC reported in the previous studies, respectively. From the tables, it can be realized that there is no specific and absolute value of rheological parameters for 3DPC, even in the state of the same mix design but different rheometer. A comparison of rheological parameters between 3DPC and self-compacting concrete (SCC) is shown in Figure 9. It is apparent that SCC has low yield stress to facilitate the passing ability and self-leveling while having high viscosity to eliminate segregation and bleeding. In contrast, 3DPC needs high yield stress to withstand the printed layer after extrusion, as well as low or pauper viscosity because the segregation is comparatively low. It should be noted that SCC has high formwork pressure, while 3DPC has the capability to carry self-weight and therefore does not require formwork [42].

Thixotropy is a time-dependent concept and property that expresses the changes in the state of pastes under shear and non-shear forces. Particularly, 3DPC must have low dynamic yield stress (good fluidity) before extrusion and high static yield stress (excellent stacking) after extrusion. In other words, 3DPC must exhibit proper structural build-up during the printing process to eliminate deformation and collapse. This time-dependent phenomenon, which is related to the structural recovery of the 3DPC, can be named thixotropy, which is one of the vital properties of this kind of concrete [57]. Ouyang and Qian et al. [58,59] proposed the I${}_{thix}$ index to explain the thixotropy of cement pastes. Based on their suggestion, thixotropy was primarily related to peak ($\tau_{0}$) and equilibrium shear stress ($\tau_{e}$). The flocculation structure may be the reason for the peak shear stress, which reduces the shear stress to the equilibrium value when the flocculation structure is broken. A larger I${}_{thix}$, which can be calculated by Equation (2), illustrates better thixotropy for cement paste.

(2) $$I_{thix}=\frac{\tau_{0}-\tau_{e}}{\tau_{e}}$$

**Figure 10:**
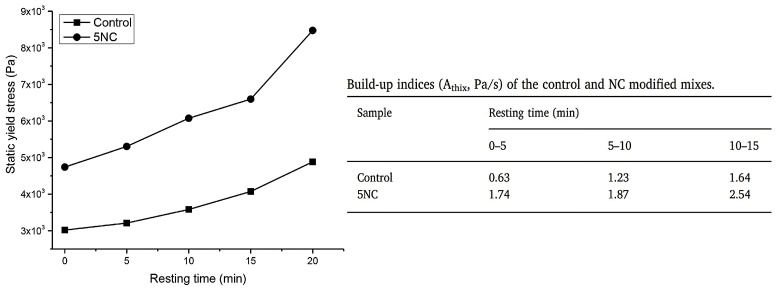
The curve of static yield stress per resting time, and the result of A${}_{thix}$. Reproduced with permission from [30].

Panda et al. [30] introduced the A${}_{thix}$ index related to thixotropy, named structural build-up. A${}_{thix}$, which is extensively employed to estimate the build-up of cementitious materials, can be calculated by quantifying the evolution of static yield stress in a resting time of 0, 5, 10, and 15 min, respectively, and the slope of the curve (Figure 10). In case of 3D-printed concrete, higher thixotropy is better because there is no formwork in this kind of concrete and each printed layer must withstand its own weight, the weight of top layer, and external forces such as nozzle pressure to prevent collapse during printing.

Jayathilakage et al. [60] published a review paper that presents comprehensive information about measuring the rheological parameters of 3D-printed concrete. They introduced a number of non-conventional methods that can be employed to investigate the rheology of 3D-printed concrete. Some of them are briefly mentioned in the following sections of this study, while others and also the details of these methods can be found in [60]. The direct shear test is a common method to evaluate the shear behavior of soil materials under normal stress. Recently, researchers employed a direct shear test in order to measure the rheological parameters of 3DPC.

With the cohesion (*C*) and the friction angle (Φ) of the tested sample, Equation (3) can be utilized to determine the correlation between the normal stress (σ) and the shear strength (τ) by considering the linear Mohr–Coulomb behavior.

(3) τ=C+σtanΦ

Researchers found that it is better for the specimens to have a low water-to-binder (w/b) ratio due to the drainage of water when applying normal stress. Therefore, they concluded that this test can be suitable for 3DPC because of the low w/b ratio in the mix design of 3DPC. It should be noted that this test can only be utilized as a static test because of the low shear rate. Hence, a flow curve cannot be attained since measurements cannot be accurate in rheometers and in high viscosity [60].

A vane shear test was used on-site to evaluate the shear strength of soil because of its ease of conduct. The lower rotational speed is the difference in this method and it can be only used to evaluate the static yield stress for cementitious material. Le et al. [61], Rahul et al. [44], and Jayathilakage et al. [28] employed this test to measure the evolution of yield stress with time (open time) for 3DPC.

### 2.2. Hardened State Properties

#### 2.2.1. Mechanical Strength

**Figure 11:**
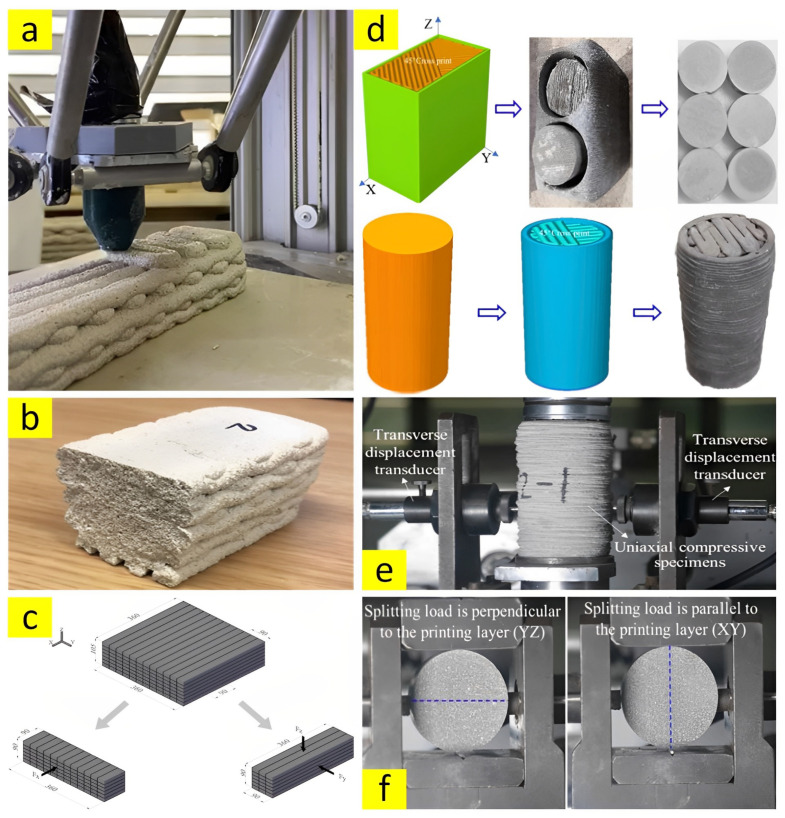
(**a**,**b**) Printed sample for compressive test, reproduced with permission from [17]; (**c**) anisotropic printed sample for flexural test, reproduced with permission from [60]; and (**d**–**f**) cylinder printed sample for compressive and tensile tests, reproduced with permission from [21].

Although previous studies used casted concrete samples to investigate the mechanical strengths of 3DPC, including compressive, flexural, and tensile strengths by following common standards such as ASTM [30], others tried to provide a situation to carry out the aforementioned tests on the printed samples [17,62]. Therefore, due to the fact that there is still no specific standard for tests carried out on the 3DPC, it is possible to pursue the common standard for casted concrete samples (such as ASTM) or accomplish the mechanical experiments on the printed specimens (such as in Figure 11). In addition, based on the previous studies, 3DPC specimens would show apparent anisotropy after hardening due to an inherent property of 3D-printed layered structures [63,64]. Accordingly, as shown in Figure 11c,f, some studies were dedicated to investigating the anisotropic behavior of 3DPC by applying load in different directions (X, Y, and Z) [65].

#### 2.2.2. Inter-Layer Bond Strength

**Figure 12:**
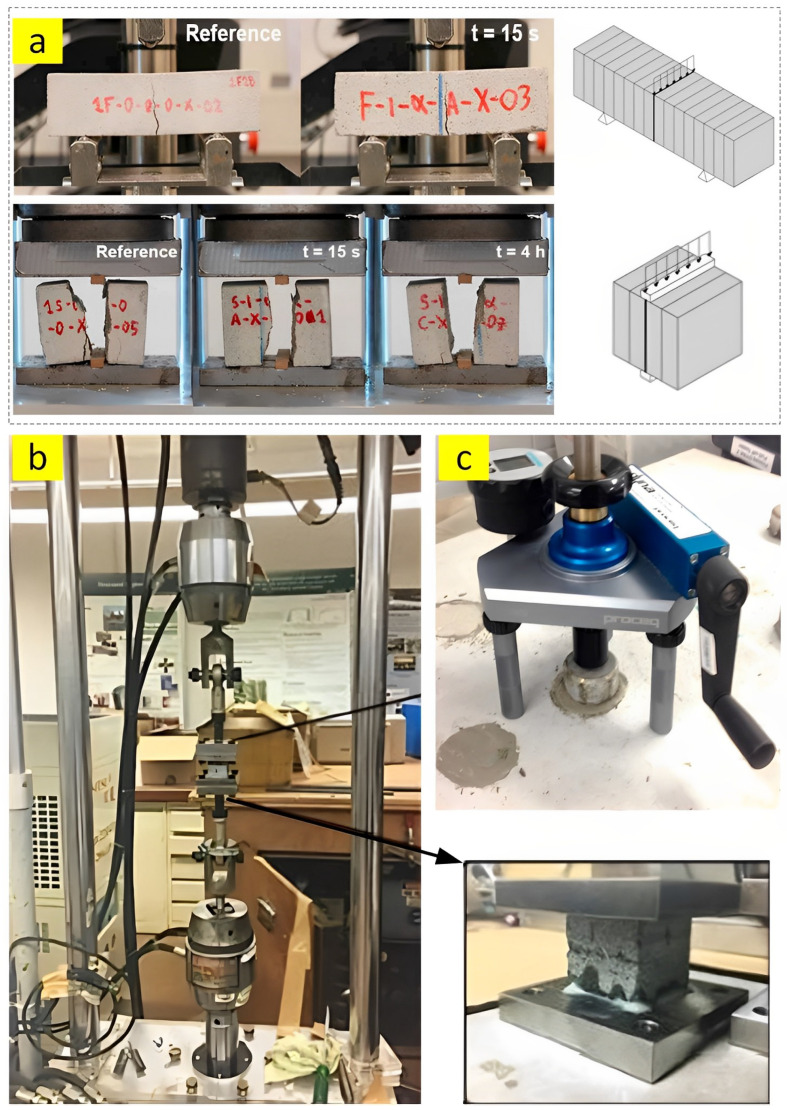
(**a**) Indirect inter-layer bond test, reproduced with permission from [64]. (**b**) Inter-layer bond test using tensile force, reproduced with permission from [20]. (**c**) Pull-off test apparatuses, reproduced with permission from [66].

The bond between the printed layers is one of the crucial factors in all additive manufacturing systems. Hence, successful evaluation of the inter-layer bond of 3D-printed concrete is necessary to warrant that there is efficient load transfer and proper bonding between the printed layers. To reach this purpose, some researchers commonly adopted the bending test by applying compression load [64] (Figure 12a), and others employed the uniaxial tensile test [20,35] (Figure 12b) to measure the inter-layer bond of printed layers. In the second method, the top surface of each layer should be effectively glued to the steel block with an adhesive agent (e.g., epoxy resin) so that the failure happens in the inter-layers when the applying tensile load. Moreover, Mohammed et al. [66] conducted a pull-off test by following the procedure of the ASTM-C1583 standard (Figure 12c). First, a 0.5 cm layer of the mixture was placed on a surface of concrete and cured for 28 days. A loading fixture with a diameter of 5 cm was glued to the samples with an adhesive agent. Finally, the specific apparatus was employed to carry out the experiment considering the 0.1 MPa/s loading rate.

#### 2.2.3. Durability

In addition to the importance of fresh state properties of 3DPC, the durability behavior of this kind of concrete should be properly investigated. Therefore, the permeability of 3DPC can be scrutinized by common experiments such as MIP (mercury intrusion porosimetry), water absorption, sorptivity, etc. The MIP test is a useful microstructural analysis in terms of volume and size of porosity in the cementitious matrix that has been employed by researchers for 3DPC [17]. This test can be effective for evaluating the pore structure of cementitious composites containing a wide range of pore diameters. Moreover, in order to investigate the 3DPC performance against water penetration in terms of both full (volumetric water absorption) and partial (capillary water absorption) immersion, the procedures of ASTM-C1585 and ASTM-C642 can be followed, respectively. It should be noted that, because there is still no specific guideline or standard for assessing the hardened properties of 3DPC, these tests can be conducted on the casted samples based on the standard or crushed printed sample by introducing new methods. Other tests on durability of cementitious composites such as chloride ion penetration and electrical resistivity can be performed with respect to the aforementioned explanation.

## 3. Mix Proportion of 3D-Printed Cementitious Composites

**Figure 13:**
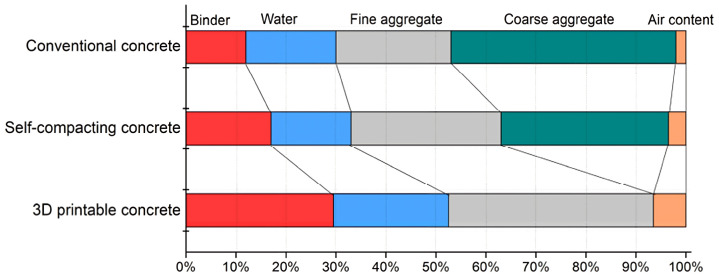
Mixture composition by percentage of volume in conventional concrete, self-compacting concrete, and 3D-printable concrete. Reproduced with permission from [42].

Most definitely, new-generation concretes have many differences from traditional concrete, including the mix design. The mix proportions of conventional concrete, self-compacting concrete, and 3D-printing concrete (3DPC) are shown in Figure 13. It is clear that the amount of coarse aggregate is one of the most remarkable differences between 3D-printing concrete and conventional concrete, in that there is no coarse aggregate in 3DPC. This can be ascribed to the fact that coarse aggregate is not printable generally, and it may be blocked during the pumping and extrusion phases, although recent studies have been trying to solve this issue by printing a mixture containing coarse aggregate [67,68,69,70,71]. Based on this reason, the amount of fine aggregate and binder in 3DPC is higher than in conventional concrete in order to increase the yield stress and the thixotropy and buildability of 3DPC. More content in the binder can effectively ameliorate the important properties of 3DPC, including pumpability, extrudability, and buildability. However, considering that the binder consists mainly of cement as an important agent for CO_2_ emissions, the question of the environment-friendliness of 3DPC technology may emerge. This issue has caused the utilization of supplementary cementitious materials (SCMs) such as silica fume, fly ash, limestone powder, and recent nanomaterials as mandatory rheology-modifying agents in the mix proportion of 3DPC in order to decrease the content of cement [72,73]. Moreover, SCMs can significantly ameliorate the fresh and hardened properties of 3D-printed cementitious composites.

In recent decades, the science of nanotechnology has extensively grown and nanomaterials have been widely utilized in compounds with many conventional materials [74,75,76]. The prominent chemical and physical properties of nanomaterials have increased the attention paid by researchers and experts active in various industries to these revolutionary materials [77,78,79]. Nanoscience has quickly entered the field of construction materials and has caused huge developments in this field [80,81,82]. Nanoparticles such as nanoclay [83,84,85,86,87,88,89,90,91,92], nanosilica [16,93,94,95,96,97,98,99,100,101], nano-Fe_2_O_3_ [102,103,104,105,106,107,108,109,110,111], nano-TiO_2_ [13,14,15,112,113,114,115,116], CNTs (carbon nanotubes) [12,117,118,119,120,121], nano-carbonate calcium [122,123,124,125,126], nano-ZnO [127,128,129], nano-Al_2_O_3_ [130,131,132,133,134,135], graphene oxide [136,137,138,139,140,141], and halloysite nanotube [9,10,142,143,144,145,146,147,148,149] have been employed to ameliorate the properties of cementitious composites. Several studies have been performed on the effects of nanomaterials on the properties of cementitious composites, and most of them report positive impacts such as improvement in mechanical properties [150], reduction in permeability [98], decrease in chloride ion penetration [151], lower water absorption [16], as well as better durability [152]. As will be presented in Section 4, a small dosage of nanomaterials can significantly affect the fresh and hardened characteristics of 3D-printing cementitious composites.

**Figure 14:**
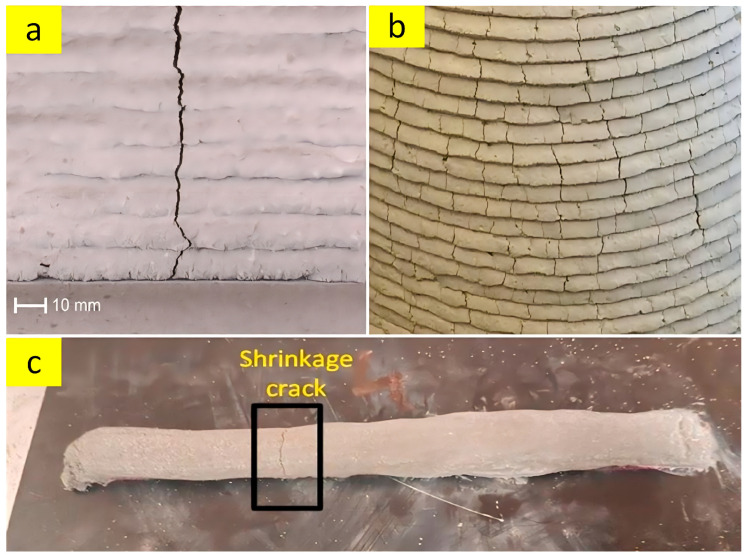
(**a**) Crack produced at a moderate rate of evaporation (2 h after printing), reproduced with permission from [160]. (**b**) Excessive cracking because of early-age shrinkage, reproduced with permission from [163] (**c**) Shrinkage crack after 24 h at laboratory temperature, reproduced with permission from [164].

Fibers are another additive material that can be utilized in order to improve the properties of 3D-printing cementitious composites. Brittle behavior, low tensile strength, and inappropriate resistance against shrinkage cracks are critical weaknesses of cementitious composites [153]. In accordance with the previous studies, suitable types and optimum dosages of fibers such as polypropylene [154], steel [155], carbon [156], glass [157], basalt [158], natural fibers [159] and synthetic fibers [160] can improve the mechanical properties of concrete (especially tensile and flexural strength), arrest the shrinkage cracks, and ameliorate durability such as the abrasion resistance of cementitious composites. Moisture loss and potential shrinkage cracks are a big challenge in 3D concrete printing due to the lack of formwork [26], the high content of fine particles, the lack of internal limitations from coarse aggregates, and the hydration process in the open atmosphere [161]. The rate of plastic shrinkage depends on several factors, including the temperature and moisture of the ambient environment, air flow, as well as the drying susceptible area. Moreover, the low water-to-binder ratio accelerates the hardening of the mixture, resulting in an increment of autogenous shrinkage [162]. Based on this reason, researchers have suggested utilizing polypropylene fiber as a shrinkage reinforcement for 3DPC mixture [26]. Examples of shrinkage cracks that occurred at early ages of 3DPC are shown in Figure 14.

According to the significant development of 3DPC in the recent decades, researchers have studied many kinds of 3D-printing special cementitious composites such as geopolymer concrete [34,51,165,166,167,168,169], underwater printable concrete [170], foam concrete [171,172,173,174,175,176,177,178], engineered cementitious composite [33,179,180,181,182], magnesium potassium phosphate cement concrete [183,184,185,186,187], and earth-based materials [188,189,190]. Although, most definitely, the mix proportion of each special concrete is different, the water–binder ratio and sand–binder ratio frequently report within the range of 0.30–0.40 and 1.2–2.0, respectively.

## 4. Effect of Nano and Micro Materials on 3DPC

### 4.1. Silica-Based Materials

Silica-based materials with a spherical shape are mainly composed of SiO_2_ and are significantly utilized in cementitious composites in both nano and micro sizes. However, silica-based nano-sized materials are used more than micro-sized ones due to their eminent properties. Nano-silica (NS) is one of the most common nano additives employed by researchers in the modification of normal cementitious composites. This fact is ascribed to its valuable physical and chemical properties, including pozzolanic activity, nucleation site effect, and filling effect, resulting in the acceleration of the process of cement hydration and formation of C-S-H gel. This performance of nano-silica provides a notable effect on the thixotropic behavior of the paste matrix as a very important factor in 3DPC. Therefore, the nano-silica can be considered a thickener for 3DPC due to its large specific surface area [191].

Mendoza et al. [192] investigated the effect of nano- and micro-silica on the properties of 3D-printable concrete. These authors found out that silica-based materials can effectively increase the static yield stress as well as the rate of thixotropic build-up of the 3D-printable cementitious composite. Nano-silica and micro-silica have a thickening effect associated with increases in water demand. Moreover, the packing density of the granular fraction [193] is another reason that micro-silica is a thickening agent, leading to an improvement in yield stress.

**Figure 15:**
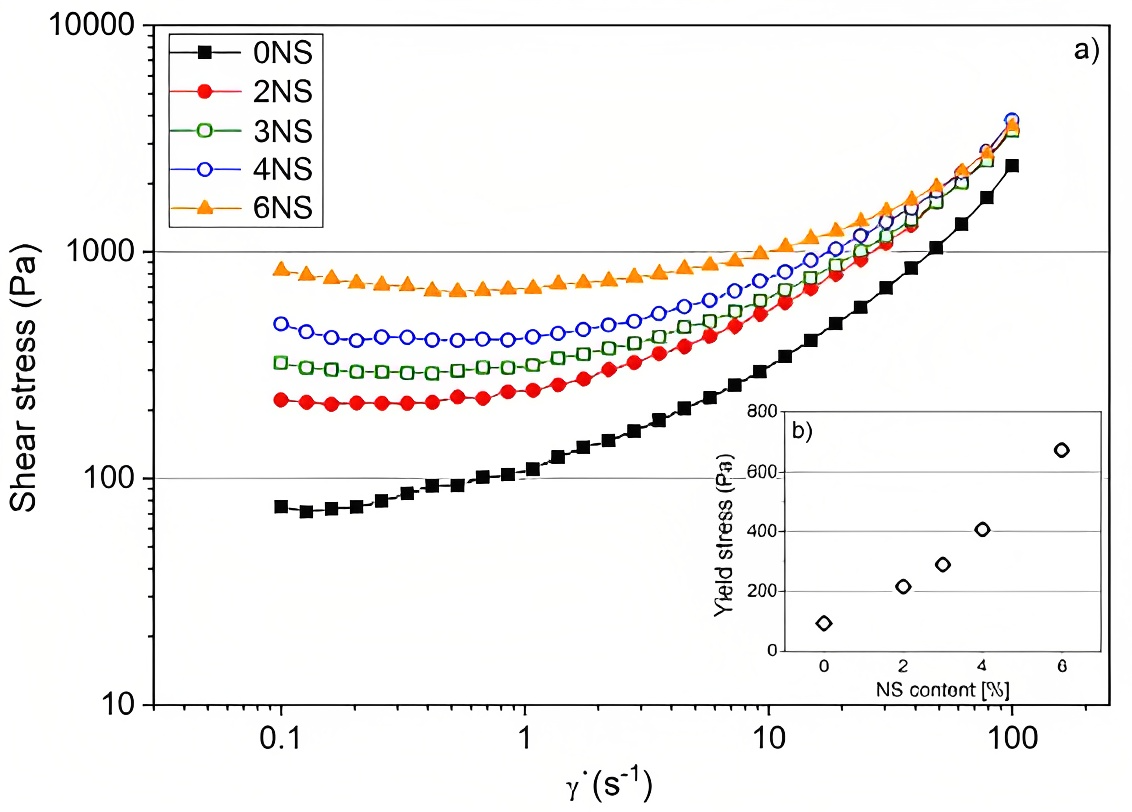
(**a**) Shear stress as a function of the shear rate of printable mortars with different NS contents, and (**b**) yield stress at 0.4 s${}^{-1}$ vs. NS dosage. Reproduced with permission from [17].

In the research of Sikora et al. [17], the effects of 2, 3, 4, and 6 wt% of nano-silica were evaluated on the fresh properties of a 3D-printable cementitious composite, specifically mortar. The results highlight the remarkable effect of nano-silica on the acceleration of the hydration process of 3D-printable mortar. Indeed, the addition of nano-silica into the mix leads to the faster formation of Ca(OH)_2_ and more consumption of C3S phases. Moreover, raising the NS content resulted in a reduction in initial and final setting times as well as a shortening of open time. Other experiments, including penetration measurements and ultrasonic tests, also confirmed that the setting process of cementitious composite is accelerated by incorporating NS into the mortar. According to Figure 15, it could be observed that nano-silica can effectively increase the yield stress of the printable mortar, which displays the increment of its thixotropy behavior. This phenomenon presents the fact that the buildability of mortar is ameliorated by the incorporation of NS, leading to printing more layers in a short time and hence speeding up the process of printing. Nevertheless, it is clear that the addition of nano-silica increases the shear stress, therefore needing a higher pumping pressure, as the pressure is proportional to the viscosity of printable cementitious composite. It should be noted that the decrease of viscosity differences with the increase of shear rate proves the fact that nano-silica modifies the mortar microstructure at rest. The phenomenon can be ascribed to stronger and better flocculation of the particles at that period in the matrix.

**Figure 16:**
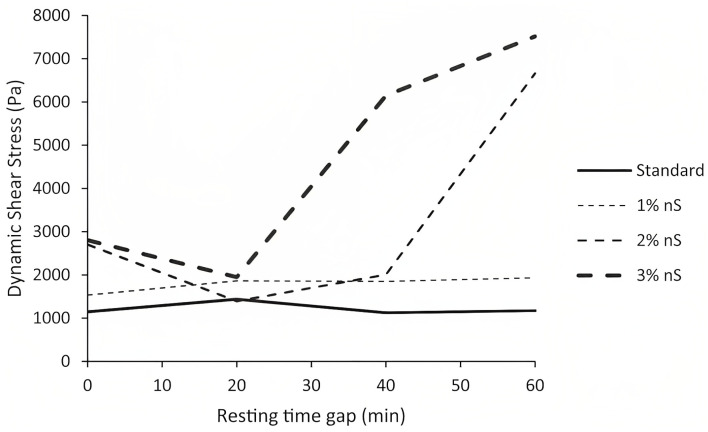
Long-term dynamic shear stress increase vs. resting time gap depending on nano-silica content. Reproduced with permission from [45].

**Figure 17:**
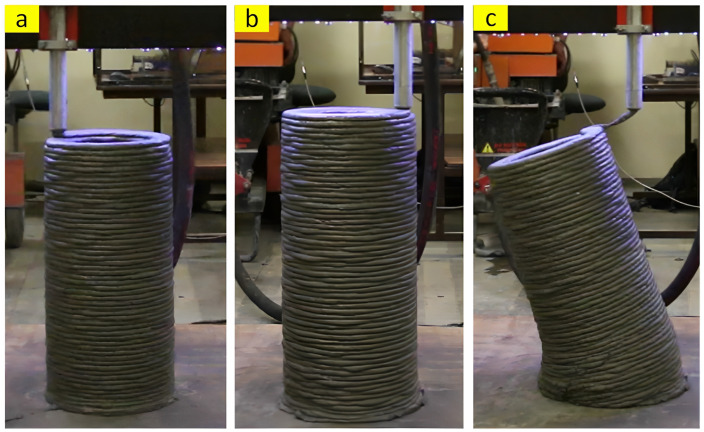
A 3D-printed circular hollow column with (**a**) standard concrete mix; (**b**) 1% NS concrete mix; (**c**) typical instability that failed. Reproduced with permission from [45].

The study conducted by Kruger et al. [45], which analyzed the effect of different dosages of nano-silica on the rheological characteristics of 3D-printing concrete, shows that nano-silica can further increase the rate of re-flocculation. The highest rate of 8 Pa/s was seen in the specimen containing 1% nano-silica, whereas higher contents of nano-silica resulted in a remarkably lower rate of re-flocculation, thus leading to a negative effect on thixotropic behavior. Moreover, the overdosage of nano-silica resulted in a remarkable increment in the long-term dynamic shear stress; hence, the mixture containing 3% nano-silica was extremely stiff and not suitable for 3D-printing concrete at the time interval of 40 min (Figure 16). It can be seen from Figure 17 that the buildability of nano-silica-modified concrete is significantly better than concrete without nano-silica. The mixture with and without nano-silica achieved a total height of 59 cm and 54 cm, respectively. Therefore, 1% nano-silica is able to increase the number of printing layers from 54 to 59 (layer height is 1 cm), resulting in accelerating the printing process and reducing the construction time.

The research of Cho et al. [171] indicates that the optimum percentage of nano-silica can effectively improve the rheological behavior and buildability of 3D-printing lightweight foam concrete. According to the results, the yield stress significantly increases with the addition of nano-silica. The incorporation of nano-silica into the mix improves re-flocculation after agitation and decreases total water content in the mix, resulting in the increase of thixotropy of printed lightweight foam concrete.

Moreover, a comparative investigation between the effect of silicon carbide nanoparticles and nano-silica on the fresh behavior of 3D-printing concrete was performed by Kruger et al. [194]. According to the results, silicon carbide improves significantly the thixotropy of 3D-printed concrete, whereas nano-silica has a noticeable effect on the static yield stress.

The effect of silica fume on the properties of 3D-printing concrete has been investigated by Rahul et al. [44]. Their results indicate that the 10% incorporation of silica fume results in enhanced robustness. Furthermore, the yield stress and buildability increase with the addition of silica fume. In addition, the time limit of printability for a sample containing silica fume estimated by Rahul et al. is 30 min.

**Figure 18:**
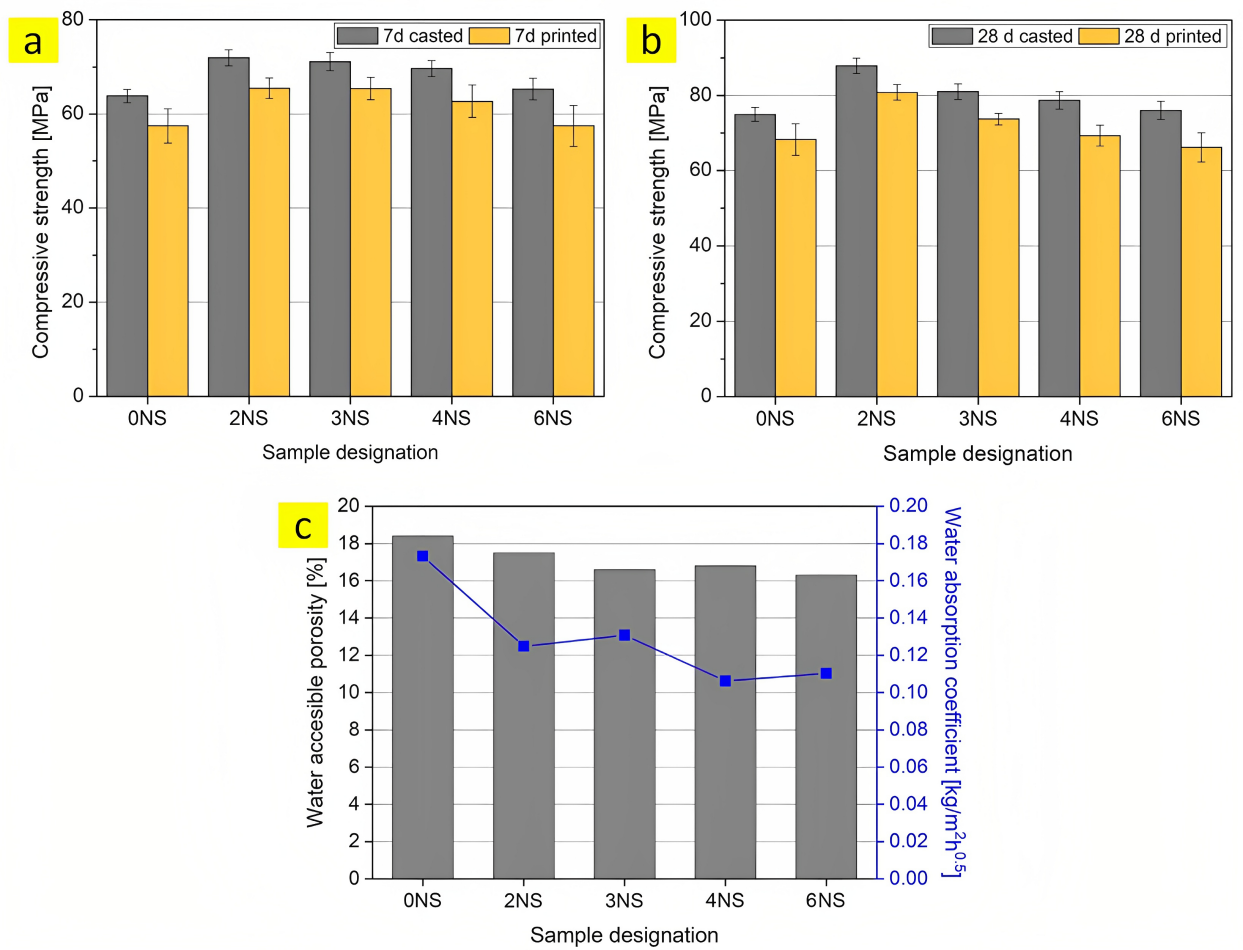
Compressive strength of casted and printed specimens after (**a**) 7 and (**b**) 28 days of curing, and (**c**) water-accessible porosity and water absorption coefficient of printable mortars. Reproduced with permission from [17].

Sikora et al. [17] investigated the effects of 2, 3, 4, and 6 wt% of nano-silica on the hardened properties of a 3D-printable cementitious composite, specifically mortar. Based on Figure 18a,b, the results of the compressive test show that the compressive strength of printed samples is lower than the casted samples by 8 to 14%. Consequently, an optimum dosage of nano-silica improves significantly the compressive strength of mortar. Furthermore, nano-silica-modified samples represented a reduction of 5 to 12% and 25 to 39% in water-accessible porosity and water absorption coefficient, respectively (Figure 18c). MIP analysis reports that nano-silica can notably ameliorate the impermeability of printable mortar by contributing to the refinement of the pore structure.

**Figure 19:**
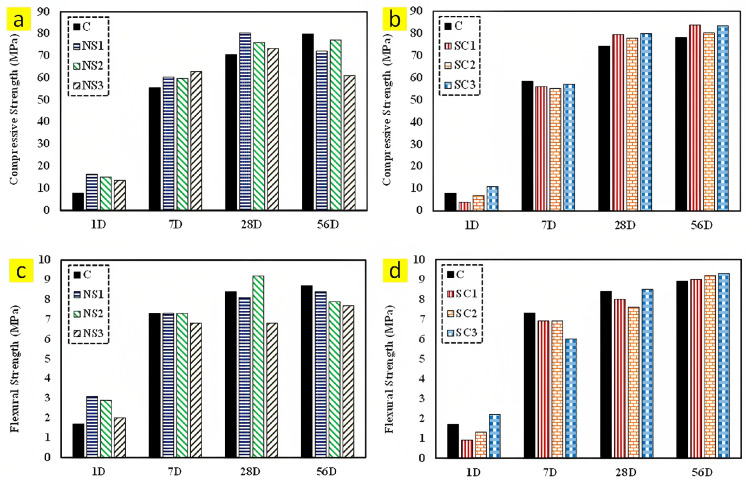
Compressive strength of sample containing (**a**) nano-silica and (**b**) silicon-carbide nanoparticles, and flexural strength of sample containing (**c**) nano-silica and (**d**) silicon-carbide nanoparticles. Reproduced with permission from [194].

The effects of nano-silica and silicon-carbide nanoparticles on the mechanical performance of 3D-printable concrete were studied by Kruger et al. [194]. It is apparent from their outcomes that the early-age compressive strength (Figure 19a,b) and flexural strength (Figure 19c,d) increase remarkably in samples containing nano-silica, whereas the development of early strength remains almost constant in the specimens with different dosages of silicon-carbide nanoparticles. The best improvement of compressive (88%) and flexural (82%) strengths belongs to the mixture containing 1% and 2% nano-silica, respectively, when compared to that of the control sample. Furthermore, the results of this research illustrate that the incorporation of silica and silicon-carbide nanoparticles ameliorates the inter-layer bond strength of the 3D-printed cementitious composite [194].

Cho et al. [171] investigated the effects of silica nanoparticles on the properties of lightweight foam concrete for 3D-printing applications. The outcomes illustrate that the appropriate dosage (2 wt%) of nano-silica is able to significantly ameliorate the compressive strength, flexural strength, and elastic modulus. Table 3 presents some of the herein-discussed relevant findings on the effects of nano- and micro-silica-based additives on the properties of 3D-printed concrete.

### 4.2. Clay-Based Materials

Another group of materials that have been proposed as agents to improve the thixotropic behavior of cementitious composites is clay, especially nanoclay. Although there are many clay-based materials, attapulgite, kaolinite, sepiolite, bentonite, and contaminated clays have been investigated as rheological modifier agents for cementitious materials [198,199,200,201,202,203,204,205,206,207] Many studies [30,40,85,204,208,209,210,211,212] carried out in this area have reported that the proper dosage of nanoclay can significantly ameliorate the thixotropy of cementitious composites. This phenomenon can be ascribed to the flocculation effect of clay nanoparticles [201,213,214,215]. On the other hand, Quanji et al. [40] found that the incorporation of nanoclay more than the proper quantity (higher than 1.3% of cement mass) decreases the thixotropy rate.

**Figure 20:**
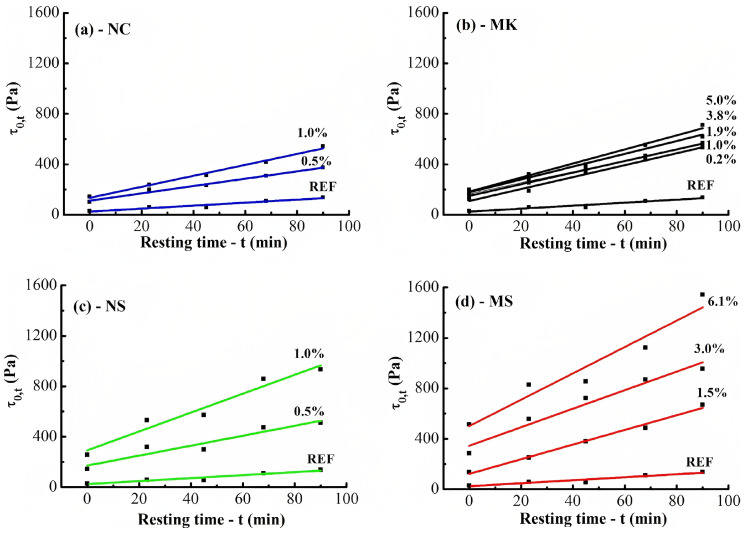
Static yield stress ($\tau_{0,t}$) vs. different resting and linear fits used to obtain A${}_{thix}$ and $\tau_{0}$, fit for pastes containing (**a**) NC, (**b**) MK, (**c**) NS, and (**d**) MS solid substitutions. Reproduced with permission from [192].

According to the research carried out by Mendoza et al. [192], as shown in Figure 20a,b, the incorporation of nanoclay and metakaolin as a thickener can acceptably increase the initial yield stress and the rate of structural build-up, which displays the improvement of thixotropy behavior of cementitious composites. The function mechanisms of metakaolin are similar to that of microsilica mentioned in the previous section in that it increases the packing density and water demand of cement matrices because of its high surface area [216]. Moreover, the thickening effect of nanoclay can be ascribed to clay particle flocculation [217], resulting from the probable interaction between ettringite and clays, or the high water absorption of nanoclay [204].

**Figure 21:**
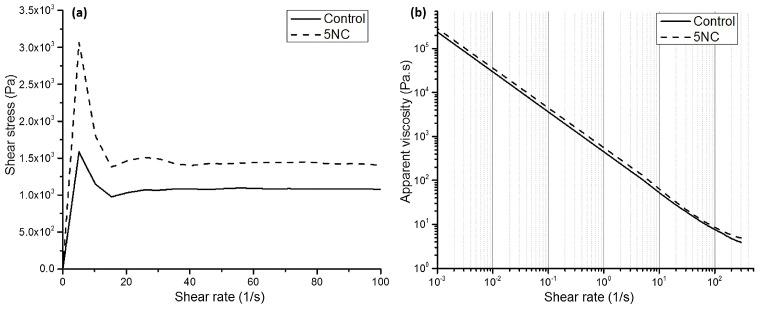
Fresh properties showing the (**a**) flow (up) curve and (**b**) shear thinning behavior of the control and NC modified mix. Reproduced with permission from [30].

Experimental results of the study published by Panda et al. [30] indicate that the incorporation of 0.5% nano-attapulgite clay increases the static yield stress of 3D-printed high-volume fly ash mortar because of the re-flocculation of clay particles and improved thixotropy, resulting in an enhancement in structural build-up at the different shear rates and resting times. Meanwhile, apparent viscosity is not significantly affected by the clay nanoparticles (see Figure 21). This behavior is strongly helpful in 3D printing concrete, where the concrete should have suitable extrudability and buildability so that can be printed layer by layer without any deformation or collapse.

**Figure 22:**
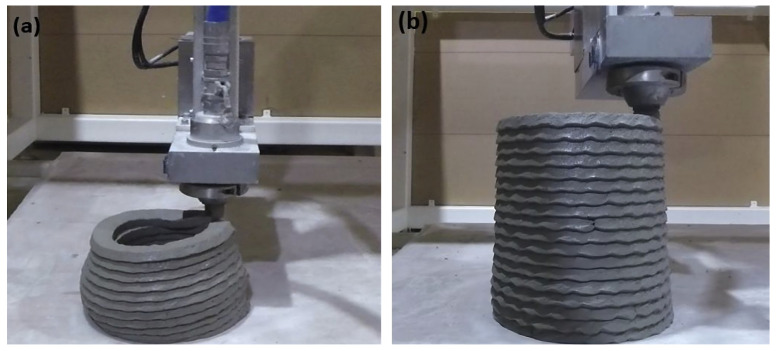
Buildability of the 3D-printable (**a**) control and (**b**) 0.5% nanoclay incorporated mixes, highlighting their bottom layer deformation. Reproduced with permission from [30].

The good buildability of mortar containing nanoclay is demonstrated in Figure 22. As can be clearly seen, the deformation of the control sample occurs in the tenth layer, whereas the nanoclay sample is able to keep its shape stability even in the twentieth layer. It should be noted that, according to previous studies, nanoclay has an immediate impact on the thixotropy properties of cementitious composites, and its effect decreases gradually with the passing of time [30,217].

**Figure 23:**
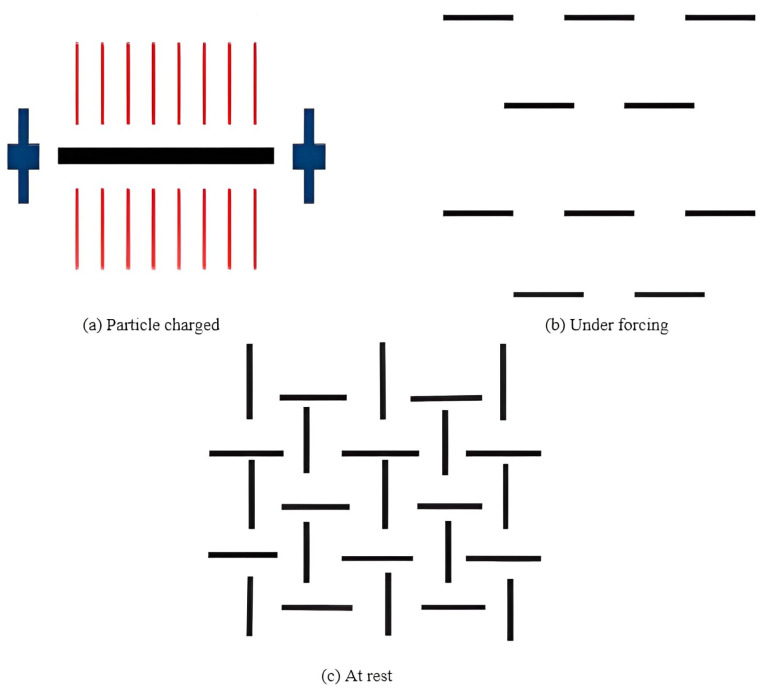
Evolution of nanoclay in the paste matrix with time. Reproduced with permission from [32].

According to Zhang et al. [32], the buildability of 3D-printed concrete is improved by about 150% with a small dosage of nanoclay, which significantly enhances the yield stress, green strength, and thixotropy without considerably affecting on viscosity. It should be noted that, based on the previous studies [40,217,218,219,220,221], nano-attapulgite clay mainly exists in needle or sheet shapes with a micron-dimension length, whereas its diameter is in the nanometer dimension. Moreover, it is negatively charged throughout the length and positively charged at the ends [40,218,220,221]. It can be seen from Figure 23b that the particles of nanoclay separate from each other under shear force. In the at-rest situation (Figure 23c), because of the van der Waals forces, the end of the clay particles with the positive charge adhere to the surface of the negative particles. This phenomenon creates a spatial network structure, resulting in providing support for the aggregate and improving the thixotropy of concrete [32,222].

Qian et al. [210] found that the addition of nanoclay improves not only the yield stress and cohesion but also the stiffness and tangent modulus. In addition to that, clay nanoparticles can decrease the critical strain by providing rigid bonding. This shows the fact that the incorporation of nanoclay ameliorates the connectivity of the microstructure of the cement matrix due to the functional mechanisms of nanoclay, including the filing effect, interparticle linkage [211], and decrease in the packing density of the cement paste system containing nanoclay [217,218].

In accordance with the research of Quanji et al. [40], the addition of 0.5–1% (by mass of cement) nano-sized highly purified magnesium aluminosilicate clay facilitates structural rebuilding (or re-flocculation of particles) and effectively increases the thixotropy of cement paste. Since the structural rebuilding of cementitious composites has an intense correlation with the rate of hydration of paste, the incorporation of nanoclay thus accelerates the rate of structural rebuilding due to the increase in the hydration rate. In another work, Kawashima et al. [204] reported the same results, where they presented that the purified attapulgite nanoclay significantly accelerates the rate of structural rebuilding, especially at early ages. Nevertheless, this phenomenon diminishes at longer resting times due to the hydration mechanisms beginning to dominate [223,224]. The effect of nanoclay on the properties of 3D-printing concrete has been studied by Rahul et al. [44]. According to the results, 0.1–0.3% nanoclay leads to enhancing the robustness. Moreover, the yield stress increases with an increase in nanoclay dosage. In general, nanoclay can effectively ameliorate the buildability of 3D-printed cementitious composites. In addition, the time limit of printability for a sample containing nanoclay estimated by Rahul et al. is 30 min.

**Figure 24:**
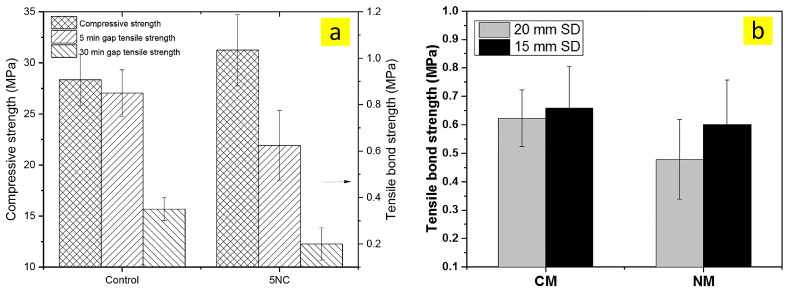
The effect of nanoclay on the (**a**) compressive and tensile bond strength, reproduced with permission from [30], and on the (**b**) tensile bond strength, reproduced with permission from [49].

According to the study of Panda et al. [30] on the mechanical performance of 3D-printed mortar consisting of high-volume fly ash (Figure 24a), 0.5% nanoclay attapulgite can increase the compressive strength by approximately 10% compared to that of the control mixture. In contrast, the addition of nanoclay leeds to a decrease in the tensile bond of strength. This can be ascribed to the thixotropy behavior of the mortar containing nanoclay, wherein the bottom layer quickly built up before the second layer was deposed over it. Similarly, in a later research, Panda et al. [49] reported that the 3D-printed concrete with nanoclay addition has lower tensile bond strength than the control sample (Figure 24b).

**Figure 25:**
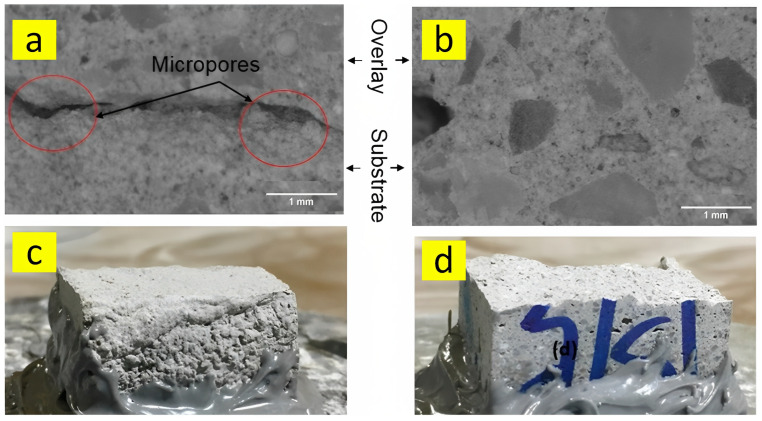
Optical microscopic images of the (**a**) nanoclay-modified sample and (**b**) control sample; fracture surfaces of the (**c**) mixture containing nanoclay and the (**d**) control mixture. Reproduced with permission from [49].

Furthermore, optical microscopic images and fracture surfaces obtained from the interface of 3D-printed samples are seen in Figure 25. The micro-pores can be clearly observed in Figure 25a, which caused the loss of the inter-layer bond strength. Accordingly, it is realized that the porosity that existed in the interface can play a vital role in the performance of the interfacial bond. Moreover, as depicted in Figure 25c,d, the nanoclay-modified sample showed brittle failure, whereas the control sample showed shearing failure because of the strong adhesion [49].

Table 4 presents some of the herein-discussed relevant findings on the effects of micro- and nanoclay-based additives on the properties of 3D-printed concrete.

### 4.3. Carbon-Based Materials

Carbon-based nanomaterials such as carbon nanotubes (CNTs), nano-graphite platelets, graphene nanoplatelets, and graphene oxide are branded as innovative and advanced additives for improving the properties of cementitious composites. The strong positive effects of this kind of nanomaterials on the fresh and hardened properties of various kinds of concretes have been proven by many researchers [152,228,229,230,231]. Graphene-based nanomaterials not only have thickening performance for increasing the thixotropy of concrete but also have remarkable mechanical performance, particularly high tensile strength and elastic modulus, which makes them a proper candidate for use in cementitious composites [232]. Given the fact that 3D concrete printing can be named a newfound technology in the construction industry, few studies have been published in the field of incorporation of graphene-based nanomaterials in 3D-printed concrete.

**Figure 26:**
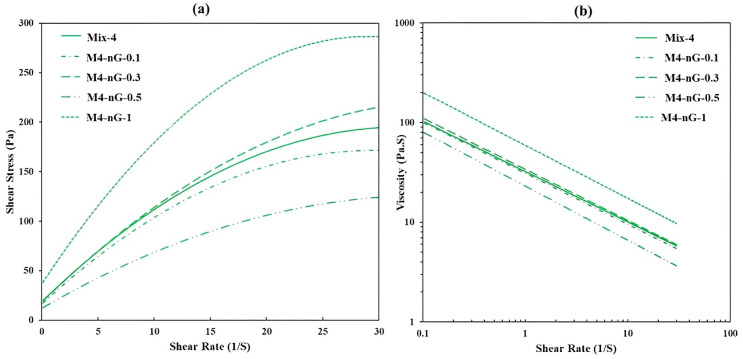
Rheology parameters of samples with different nano-graphite content, (**a**) shear stress, and (**b**) viscosity. Reproduced with permission from [19].

The effects of nano-graphite platelets on the properties of 3D-printed multi-binder geopolymer were investigated by Chougan et al. [19] (Figure 26). According to the outcomes, the fresh properties of geopolymer had been ameliorated by the incorporation of nanomaterials in specific amounts (i.e., 0.3% and 1% by the weight of the binder) when compared to that of the control specimens. The samples containing 1% nano-graphite demonstrate the best improvement among all mixtures, where adding nanomaterials enhanced the yield stress and plastic viscosity by about 96% and 56%, respectively, in comparison with the control mix. Moreover, the addition of 1% nano-graphite improved the shape retention of geopolymer by up to 50%. This phenomenon can be ascribed to the Van Der Waals forces between graphite nanoparticles and the super sorbent characteristics of this kind of nanomaterials, leading to a decrease in the space between the main components of geopolymer and better buildability. In addition to that, the presence of such nanomaterials can make the paste more cohesive, resulting in a denser microstructure and better shape retention [19]. Based on the results obtained from shape retention and rheology tests, the higher the viscosity and yield stress, the better the shape retention [19,213]. In another research, Zhong et al. [233] reported that the inclusion of graphene oxide nanoparticles ameliorates the rheology performance of geopolymer, which improves the extrudability and buildability of geopolymer.

**Figure 27:**
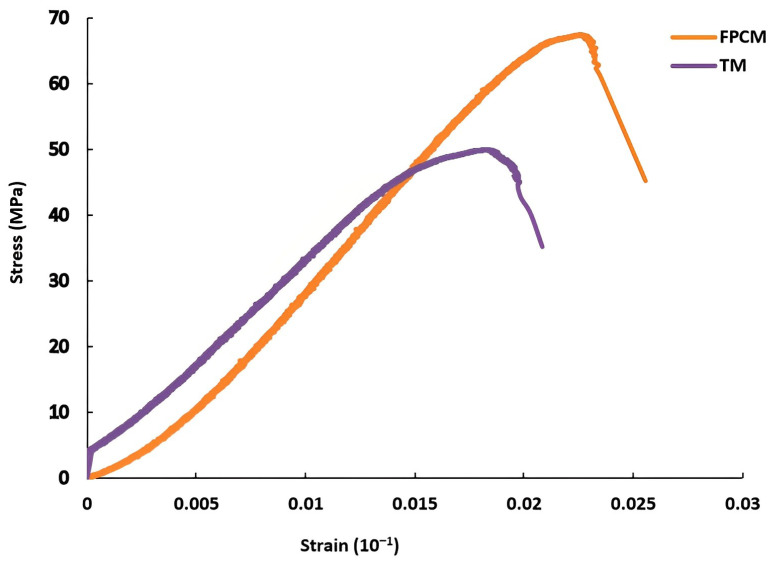
Stress−strain behavior of a 3-day sample with graphene oxide (FPCM) and a 28-day sample without graphene oxide (TM). Reproduced with permission from [66].

The effects of graphene oxide on the properties of the ultra-high early-strength cementitious composite were studied by Alyaa et al. [66]. The outcomes of this research illustrate that graphene oxide remarkably contributes to improving the mechanical properties of the 3D-printed cementitious composite, especially tensile strength, failure strain, and inter-layer bond strength. Furthermore, the layers of graphene nanoparticles play a vital role in eliminating the propagation of microcracks in the matrix, leading to an increase in the capacity and ductility of the cementitious composite (Figure 27) [66].

Furthermore, the prospect of carbon nanotube (CNT) application and development as a potential additive in 3D-printed concrete has also been considered. The presence of CNT in the mixture of 3DPC can accelerate the setting time and ameliorate production efficiency. It can effectively provide a 3DPC mixture with lower drying shrinkage, better printing quality, and higher early strength [234]. Dulaj et al. [235] studied the effect of multi-walled carbon nanotubes (MWCNTs) on the properties of 3D-printed concrete and found that the incorporation of CNT can effectively improve both fresh and hardened characteristics of 3DPC.

According to the research published by Kan et al. [236], incorporation of 0.05% MWCNTs enhances the mechanical properties of the printed reactive powder concrete (RPC) by filling the pores inside the samples and bridging the effect in cracks. Moreover, a dosage of 0.05% MWCNTs is able to change the brittle failure of the printed RPC to ductile failure. In addition to that, MWCNTs remarkably reduce the porosity of the samples.

### 4.4. Other Nanomaterials

There are some micro- and nanomaterials such as nano-TiO_2_, nano-CaCO_3_, cellulose nanocrystals (CNC), and limestone which cannot be categorized into the groups mentioned in the previous sections. Therefore, this section is dedicated to investigating the impact of these kinds of materials in order to present comprehensive information including a wide range of materials. TiO_2_ nanomaterial is well known because of its photocatalytic effect that can create a cleaner 3DPC, in addition to its other effects on the fresh and hardened properties of 3DPC. Nano-carbonate calcium (nano-CaCO_3_) and cellulose nanocrystals (CNC) are other new nanomaterials which attract the attention of researchers to employ them to achieve improved performance of 3DPC due to its positive influences. Styrene-butadiene rubber (SBR) is another new potential additive that recent studies found can remarkably improve the extrudability, open time, compressive strength, and flexural strength of 3DPC.

**Figure 28:**
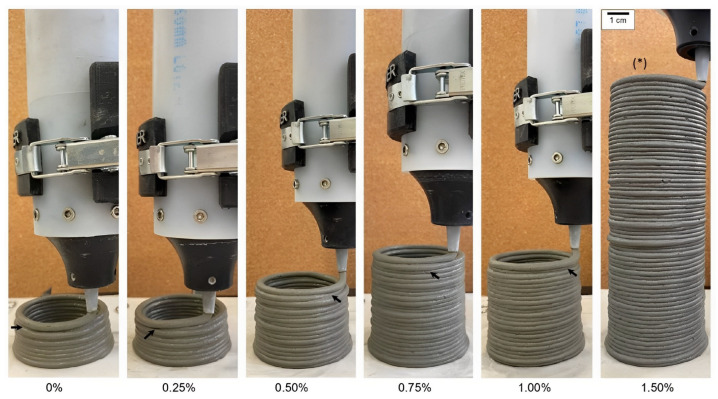
Buildability of the samples containing a different dosage of nano-TiO_2_. Reproduced with permission from [237].

The study of the effect of TiO_2_ nanoparticles on the fresh properties of 3D-printed cementitious composites was conducted by Matos et al. [237]. From the results, nano-TiO_2_ in cement composites ameliorate the cement hydration, resulting in further ettringite formation. Moreover, nano-TiO_2_ progressively increases the static and dynamic yield stress, plastic viscosity, and structural build-up. Furthermore, as can be seen from Figure 28, 1.5% nano-TiO_2_ effectively improves the buildability of the cementitious composites, leading to more successive printed layers (64 layers) when compared to that of the control sample (11 layers). In contrast, the addition of nano-TiO_2_ causes a reduction in printability from 140 min (0% TiO_2_) to 90 min (1.5% TiO_2_). According to the results, it can be concluded that, although adding “high” contents of nano-TiO_2_ (e.g., >1 wt.%) are useful for buildability, it would require a faster 3DP process. The usage of nano-TiO_2_ dosages of around 0.75–1.00% may be a reasonable selection because it reduces the printability of the mixture by 30 min but increases the buildability by around 118% (24 printed layers) when compared to the control mix.

Concerning thixotropy behavior, nano-TiO_2_ was employed by Liu et al. [238] in order to introduce a new 3D-printable cement-based composite. They observed that nano-TiO_2_-3DPC presents better thixotropy behavior and accelerates the setting time. Moreover, the proper dosage of this kind of nanomaterial can increase the static yield stress and simultaneously decrease the viscosity and dynamic yield stress. A sample containing 3% nano-TiO_2_ produces better buildability and density (19.6%↑) when compared with the undoped nano-TiO_2_ group.

Focusing on hardened properties, Liu et al. [238] reported that the addition of 3% nano-TiO_2_ increases the compressive strength by around 52% compared to the control sample. Furthermore, XRD and SEM results revealed that the nano-TiO_2_ can efficiently refine the pore structure and provide a denser microstructure for the mixtures. Nano-TiO_2_ also presented an apparent self-cleaning performance for 3D-printed cement-based samples, which demonstrates that this kind of nanomaterial is an ideal additive for clean production in civil construction.

Considering another nanomaterial of interest, Yang et al. [239] reported the impact of nano-calcium-carbonate on the properties of 3D-printed cementitious composites containing limestone powder. They used 0, 1, 2, and 3% nano-carbonate-calcium (nano-CaCO_3_) as a partial replacement for limestone powder, respectively. The nucleation effect of nano-CaCO_3_ resulted in the acceleration of the hydration reaction of the cement matrix. Moreover, NC remarkably enhanced fresh state performances of 3DPC, including yield stress, green strength, and vertical displacement through its large specific surface areas.

In the research conducted by Yang et al. [239], however, mixtures containing 15% limestone powder illustrated lower compressive and flexural strength than that of the control mix at all ages; the addition of nano-CaCO_3_ compensated for this weakness.

**Figure 29:**
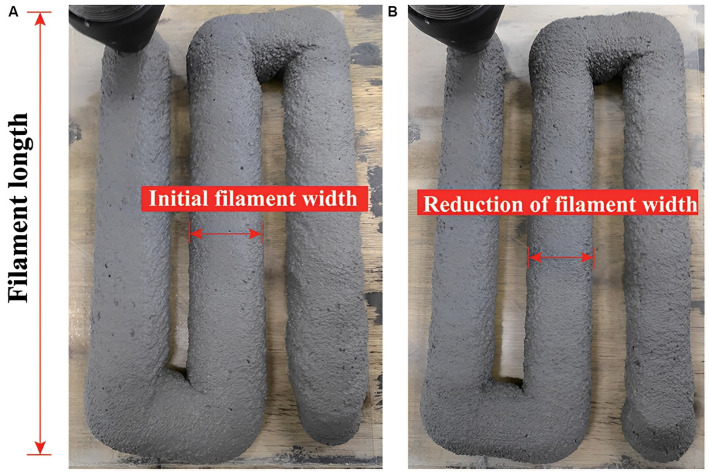
The extrudability and buildability of the 3D-printed concrete containing nano-CaO_3_. Extrusion with a 20-mm-diameter round nozzle: (**A**) initial filament width, and (**B**) reduction of filament width. Reproduced with permission from [240].

In another research, Yang et al. [240] investigated the effect of 1–4% nano-CaO_3_ on the workability, strength, and microstructure of 3D-printed cementitious composites. The large specific surface area of nano-CaO_3_ results in a reduction in fluidity, printability limit, extrudability, and self-weight deformation (Figure 29).

Furthermore, the research published by Yang et al. [240] showed that incorporating 2% nano-CaO_3_ ameliorates the compressive strength by approximately 7.2, 39.1, and 22.5% compared to that of the control mixture at 7, 28, and 90 days, respectively, due to the seeding effect of this nanomaterial. Moreover, the filling effect of nano-CaO_3_ caused the denser microstructure of the 3D-printed mixture.

Ghantous et al. [241] studied the impact of cellulose nanocrystals (CNCs) on the drying behavior of 3D-printed cementitious composite through neutron radiograph. The results illustrated that the drying and degree of hydration of the samples containing CNCs were statistically similar to the samples without CNCs. Moreover, Valadez et al. [242] reported that the incorporation of CNCs decreases extrusion pressure and increases the flexural strength and the degree of hydration of the 3D-printed cementitious composite.

Table 5 presents some interesting findings on the effects of other additives on the properties of 3D-printed concrete.

## 5. Nanomaterials: A Suitable Solution for the Challenges of 3DPC

According to previous studies, thixotropy is a crucial and challenging property of 3DPC that should be improved by proper methods. From the results, it can be realized that nanomaterials can act as thickening agents and effectively overcome this challenge of 3DPC through a number of mechanisms such as the flocculation effect, acceleration in hydration, filling effect, and nucleation effect, leading to excellent rheological and fresh characteristics for 3D-printed concrete. This phenomenon can compensate for the buildability weakness as another vital challenge of 3DPC. Observations proved that the printing height of samples containing nanomaterials is remarkably higher than the control sample. In terms of the hardened performance of 3DPC, weak flexural and inter-layer bond strengths in addition to high porosity, especially in the zone of two printed layers, are the obvious challenges that must be solved. One- (e.g., carbon nanotube) and two-dimensional (e.g., graphene nanoplate) nanomaterials can bridge nano- and micro-cracks in the matrix and increase the flexural and tensile strength of 3DPC. Moreover, the mechanisms mentioned above result in denser microstructure and lower porosity in the 3DPC matrix. Limited studies reported that nanomaterials can also improve the inter-layer bond strength of 3DPC. However, a deep understanding of the effect mechanism of nanomaterials on the inter-layer bond strength of 3DPC is a crucial issue that should be studied in future research. Nonetheless, it can be seen that nanomaterials exhibit great potential to solve the important challenges of 3DPC to achieve 3D-printed concrete with high performance.

## 6. Conclusions

The recent research on the main characteristics of 3D-printed concrete and the influence of nano and micro additives on the performance of 3DPC are reviewed in the current paper. Our conclusions can be presented as follows:

- Some fresh state experiments including flow table, slump, setting time, and open time are employed by researchers to evaluate the printability of the 3DPC mixture before starting the printing process. Moreover, the performance of the mixture during the printing stage is analyzed by extrudability and buildability tests. Shape stability and green strength tests can be employed to evaluate the properties of 3DPC in the stage after printing but still in a fresh state. Furthermore, rheology is a critical experiment for this kind of concrete that reports three main data including yield stress, viscosity, and thixotropy.
- Although there is still no specific standard for evaluating the properties of 3DPC, mechanical strength, inter-layer bond strength, and some durability tests such as mercury intrusion porosimetry (MIP), sorptivity, and water absorption were conducted by recent studies to investigate the hardened state performance of 3DPC.
- In terms of fresh state properties, nanomaterials can be mainly considered a thickener for 3DPC due to their large specific surface area, thus ameliorating the thixotropic behavior and the structural build-up of 3DPC. The incorporation of nanomaterials such as nano-silica, nanoclay, nano-TiO_2_, nano-CaCO_3_, and graphene increases the number of printed layers (buildability) without any deformation due to their high flocculation rate. Higher yield stress and viscosity recovery can be observed in the samples containing nanomaterials compared to that of the reference samples.
- Recent studies proved that nanomaterials can notably ameliorate the hardened performance of 3DPC. This kind of material can effectively fill out the pores existing in the mixture matrix due to their nano-scale size and produce additional C-S-H gel by their nucleation effect, resulting in denser microstructure, lower permeability, and higher mechanical strengths, especially compressive strength and inter-layer bond. In addition to that, each nano additive has its own impact affecting the mixture. Nano-silica is categorized as a super-pozzolanic material so that can change the extra Ca(OH)_2_ of the matrix to new C-S-H and help to make the matrix denser. Tubular nanomaterials such as carbon nanotube (CNT) can bridge the nano-cracks in the matrix because of their high length-to-diameter ratio (similar to the role of reinforcement and fiber in the macro and micro scale, respectively), and in this way, they improve flexural and tensile strength. The swelling property of clay-based nanomaterials can decrease the pore volume of 3DPC. Nano-TiO_2_ also presented apparent self-cleaning behavior for 3D-printed concrete due to its photorealistic effect.
- It should be noted that the positive influences of nanomaterials happened in their proper dosage. Therefore, extra dosages (higher than optimal) can negatively affect the fresh and hardened properties of 3DPC.
- Supplementary cementitious materials such as silica fume, metakaolin, and limestone powder can be also named thickening agents to improve the fresh properties of 3DPC. They effectively diminished the volume bleeding rate and caused higher plastic viscosity, static and dynamic yield stress, buildability, and green strength, and the presence of bentonite in the mixture also resulted in better compressive strength of 3DPC. However, it can be concluded that the impact of nanomaterials on the performance of 3DPC is considerably higher than that of micromaterials. Moreover, silica-based additives illustrated better results when compared to clay-based additives. Styrene-butadiene rubber (SBR) is another new additive that some researchers reported can remarkably ameliorate extrudability, open time, compressive strength, and flexural strength while slightly decreasing buildability.
- According to the reviewed studies, it can be concluded that, despite acceptable investigation of the fresh properties of 3DPC reinforced with nanomaterials, more research should be carried out to evaluate in depth the hardened properties of 3DPC, especially in terms of durability. In addition to that, researchers should devote more research to investigating the effects of the above-discussed additives on the performance of other kinds of 3DPC such as lightweight 3DPC, self-compacting, etc. A deep understanding of the effect mechanism of nanomaterials on the inter-layer bond strength of 3DPC is another vital issue that should be considered in future studies. Moreover, nano-scale fillers from source of waste and biomass can be attractive additives for future research in order to achieve high performance of sustainable 3D-printed concrete.

## Figures and Tables

**Table 1:** Static yield stress reported in previous studies for different 3DPC [42].

Concrete Type	Reference	Testing Apparatus	Static Yield Stress (MPa)
Cement-based mortar	Perrot et al. [43]	Anton Paal Rheolab rheometer	4
Cement-SCM * blended mortar	Le et al. [24]	Shear vane test	0.3–0.9
	Rahul et al. [44]	Shear vane test	1.5–2.5
	Kruger et al. [45]	ICAR rheometer	2.7–3.9
	Kruger et al. [46]	ICAR rheometer	1.9
	Papachristoforou et al. [47]	ICAR rheometer	0.5–1.8
	Weng et al. [48]	Viskomat XL	3.3
	Panda et al. [49]	Anton Par MCR 102 rotational rheometer	3.2–6.8
	Moeini et al. [50]	Anton Paar MCR 302 rheometer	0.2–0.7
Geopolymer mortar	Panda and Tan [51]	Anton Par MCR 102 rotational	0.4–1
Cement paste	Chen et al. [52]	Rotational rheometer	0.2–0.7

* SCM: supplementary cementitious materials.

**Table 2:** Dynamic yield stress and plastic viscosity reported in previous studies for different 3DPC [42].

Concrete Type	Reference	Testing Apparatus	Dynamic Yield Stress (MPa)	Plastic Viscosity (Pa·s)
Cement-SCM blended mortar	Moeini et al. [50]	Anton Paar MCR 302	0.1	1.9
	Zhang et al. [53]	-	0.1–0.2	3.5–4.1
	Jayathilakage et al. [54]	Rotational rheometer	1.2–1.8	24.2–47.1
Printable ECC *	Zhu et al. [33]	Brookfield RST-SST	0.2–0.5	3.7–11.7
Cement paste	Nair et al. [55]	Dynamic shear rheometer	0.1–0.3	1.6–4.2
	Chen et al. [52]	Rotational rheometer	0.5–0.6	2.4–2.6
	Chen et al. [56]	Rotational rheometer	0.5–0.7	2.4–2.9
	Chen et al. [57]	Rotational rheometer	0.6–0.7	2.2–3.4

* EEC: engineered cementitious composites.

**Table 3:** Effects of silica-based additives on the properties of 3D-printed concrete.

**Research**	**Material**	Dosage *	**Property**	**Result (Effect of Desired Material)**
Ranjan et al. [194]	Nano-silica	4%	Fresh	Continuous growth in the plastic viscosity, power-law exponent, yield stress, and consistency coefficient by the addition of 4% nanosilica in 3DPC.
Jiang et al. [195]	Nano-silica	0.5 and 1%	Fresh and Hardened	Acceleration of setting time, reduction in fluidity, improvement in printability and homogeneity, higher compressive strength, and denser microstructure with less porosity.
Liu et al. [173]	Silica fume	6, 10, and 16%	Fresh	Silica fume effectively diminished the volume bleeding rate and increased the wet density of foam concrete. The higher the dosage of silica fume, the higher the plastic viscosity and the static and dynamic yield stress.
Srinivas et al. [196]	Silica fume	5–10%	Fresh and Hardened	The addition of silica fume reduced the anisotropic mechanical properties (71% reduction in anisotropic factor) in the printed samples. Better static yield stress and buildability of specimens containing silica fume.
Zhang et al. [32]	Silica fume	2%	Fresh	The buildability of 3D-printed concrete is improved by about 117% with a small dosage of silica fume, and significantly enhances the yield stress, thixotropy, and green strength, without considerably affecting the viscosity.
Van den Heever et al. [197]	Nano-silicon-carbide	2%	Fresh and Hardened	The incorporation of 2 wt.% silicon carbide nanoparticles into the mixture enhances the thixotropy and increases the inter-layer bond strength of the cementitious composite, resulting in a significant improvement in printability.
Zhang et al. [53]	Nanoclay and silica fume	2% of each material	Fresh	High thixotropic 3D-printed concrete with up to 129.8% improvement in the yield stress.

* By the weight of the binder.

**Table 4:** Effects of clay-based additives on the properties of 3D-printed concrete.

Research	Material	Dosage *	Property	Result (Effect of Desired Material)
Chen et al. [52]	Metakaolin	0–3%	Fresh	Thixotropy, plastic viscosity, dynamic yield stress, static yield stress, and structural deformation were well ameliorated by the incorporation of metakaolin into the 3D-printed calcium sulfoaluminate cement composites.
Chen et al. [57]	Bentonite	0–3%	Fresh and Hardened	The incorporation of bentonite into the 3D-printed calcium sulfoaluminate cement composites resulted in better thixotropy, plastic viscosity, dynamic yield stress, static yield stress, structural deformation, and compressive strength.
Douba et al. [225]	Nanoclay	4%	Fresh	Incorporation of 4.0 wt.% nanoclay increased the static yield stress (due to ionic forces) and viscosity of cement paste by around 150%, and 90%, respectively. Nanoclay could ameliorate the stiffening of the fresh state through seeding and provide high buildability with stable layer deposition.
Ramakrishnan et al. [209]	Nanoclay	0.2%	Fresh	The presence of nanoclay increased the yield strength of 3D-printed concrete from 3.5 kPa to 8 kPa at 15 min with good extrudability.
Aydin et al. [226]	Nano-montmorillonite and Sepiolite	0.5 and 1%	Fresh	Better thixotropic behavior, higher dynamic and static yield stress, no significant impact on viscosity, higher structural build-up but no effect on the structural recovery, and better printability.
Kaushik et al. [227]	Nanoclay		Fresh	Decrease in workability, increase in yield stress, cohesiveness, and stability, better shape stability, and buildability.
Zhang et al. [53]	Nanoclay and silica fume	2% of each material	Fresh	High thixotropic 3D-printed concrete with up to 129.8% improvement in the yield stress.

* By the weight of the binder.

**Table 5:** The effect of other additives on 3D-printed concrete.

Research	Material	Dosage *	Property	Result (Effect of Desired Material)
Srinivas et al. [196]	Limestone	5–10%	Fresh and Hardened	The addition of limestone powder reduced the anisotropic mechanical properties (68% reduction in anisotropic factor) in the printed samples and increased water demand. There resulted higher structural build-up, flocculation rate, and buildability of specimens containing limestone powder.
Dey et al. [243]	Limestone	5, 10, and 15%	Fresh and Hardened	Mixes containing limestone illustrated lower slump, higher yield stress and buildability, and lower inter-layer bonds.
Kim et al. [244]	Styrene butadiene rubber (SBR)	5, 10, 15, and 20 **	Fresh	Better extrudability, a slight decrease in buildability, and a remarkable increase in open time.
Branch et al. [245]	Styrene butadiene rubber (SBR)	0.05–0.2 **	Hardened	Higher compressive and flexural strengths.

* By the weight of the binder. ** SBR/cement ratio.

## Data Availability

Data sharing not applicable.

## References

[1] Onyelowe K.C., Gnananandarao T., Ebid A.M., Mahdi H.A., Ghadikolaee M.R., Al-Ajamee M. (2022). Evaluating the compressive strength of recycled aggregate concrete using novel artificial neural network. Civ. Eng. J..

[2] Khan S.A., Koç M., Al-Ghamdi S.G. (2021). Sustainability assessment, potentials and challenges of 3D printed concrete structures: A systematic review for built environmental applications. J. Clean. Prod..

[3] Jiang X., Xiao R., Bai Y., Huang B., Ma Y. (2022). Influence of waste glass powder as a supplementary cementitious material (SCM) on physical and mechanical properties of cement paste under high temperatures. J. Clean. Prod..

[4] Yang L., Sepasgozar S.M., Shirowzhan S., Kashani A., Edwards D. (2023). Nozzle criteria for enhancing extrudability, buildability and interlayer bonding in 3D printing concrete. Autom. Constr..

[5] Panda B., Tay Y.W.D., Paul S.C., Tan M.J. (2018). Current challenges and future potential of 3D concrete printing: Aktuelle Herausforderungen und Zukunftspotenziale des 3D-Druckens bei Beton. Mater. Werkst..

[6] Wangler T., Roussel N., Bos F.P., Salet T.A., Flatt R.J. (2019). Digital concrete: A review. Cem. Concr. Res..

[7] Zhou Y., Jiang D., Sharma R., Xie Y.M., Singh A. (2023). Enhancement of 3D printed cementitious composite by short fibers: A review. Constr. Build. Mater..

[8] Weng Y., Li M., Ruan S., Wong T.N., Tan M.J., Yeong K.L.O., Qian S. (2020). Comparative economic, environmental and productivity assessment of a concrete bathroom unit fabricated through 3D printing and a precast approach. J. Clean. Prod..

[9] Ghadikolaee M.R., Korayem A.H., Sharif A., Liu Y.M. (2021). The halloysite nanotube effects on workability, mechanical properties, permeability and microstructure of cementitious mortar. Constr. Build. Mater..

[10] Razzaghian Ghadikolaee M., Habibnejad Korayem A., Ghoroqi M., Sharif A. (2018). Effect of halloysite nanotubes on workability and permeability of cement mortar. Modares Civ. Eng. J..

[11] Bahari A., Sadeghi-Nik A., Shaikh F.U.A., Sadeghi-Nik A., Cerro-Prada E., Mirshafiei E., Roodbari M. (2022). Experimental studies on rheological, mechanical, and microstructure properties of self-compacting concrete containing perovskite nanomaterial. Struct. Concr..

[12] Cerro-Prada E., Pacheco-Torres R., Varela F. (2020). Effect of multi-walled carbon nanotubes on strength and electrical properties of cement mortar. Materials.

[13] Cerro-Prada E., García-Salgado S., Quijano M.Á., Varela F. (2018). Controlled synthesis and microstructural properties of sol-gel TiO2 nanoparticles for photocatalytic cement composites. Nanomaterials.

[14] Cerro-Prada E., Manso M., Torres V., Soriano J. (2016). Microstructural and photocatalytic characterization of cement-paste sol-gel synthesized titanium dioxide. Front. Struct. Civ. Eng..

[15] Cerro Prada M.E., Torres Costa V., Manso Silván M. (2016). Sol-gel TiO_2_ nanoparticles prompt photocatalytic cement for pollution degradation. Adv. Mater. Sci..

[16] Razzaghian Ghadikolaee M., Mirzaei M., Habibnejad Korayem A. (2021). Simultaneous effects of nanosilica and basalt fiber on mechanical properties and durability of cementitious mortar: An experimental study. Can. J. Civ. Eng..

[17] Sikora P., Chung S.Y., Liard M., Lootens D., Dorn T., Kamm P.H., Stephan D., Abd Elrahman M. (2021). The effects of nanosilica on the fresh and hardened properties of 3D printable mortars. Constr. Build. Mater..

[18] Chougan M., Ghaffar S.H., Jahanzat M., Albar A., Mujaddedi N., Swash R. (2020). The influence of nano-additives in strengthening mechanical performance of 3D printed multi-binder geopolymer composites. Constr. Build. Mater..

[19] Chougan M., Ghaffar S.H., Sikora P., Chung S.Y., Rucinska T., Stephan D., Albar A., Swash M.R. (2021). Investigation of additive incorporation on rheological, microstructural and mechanical properties of 3D printable alkali-activated materials. Mater. Des..

[20] Chu S., Li L., Kwan A. (2021). Development of extrudable high strength fiber reinforced concrete incorporating nano calcium carbonate. Addit. Manuf..

[21] Liu Q., Jiang Q., Huang M., Xin J., Chen P., Wu S. (2022). Modifying effect of anionic polyacrylamide dose for cement-based 3DP materials: Printability and mechanical performance tests. Constr. Build. Mater..

[22] Razzaghian Ghadikolaee M., Mirzaei M., Habibnejad Korayem A. (2021). Experimental studies of workability, mechanical behavior and durability properties of basalt-polypropylene fibers-reinforced cementitious mortar. Modares Civ. Eng. J..

[23] Bos F., Wolfs R., Ahmed Z., Salet T. (2016). Additive manufacturing of concrete in construction: Potentials and challenges of 3D concrete printing. Virtual Phys. Prototyp..

[24] Le T.T., Austin S.A., Lim S., Buswell R.A., Gibb A.G., Thorpe T. (2012). Mix design and fresh properties for high-performance printing concrete. Mater. Struct..

[25] Ma G., Li Z., Wang L. (2018). Printable properties of cementitious material containing copper tailings for extrusion based 3D printing. Constr. Build. Mater..

[26] Kazemian A., Yuan X., Cochran E., Khoshnevis B. (2017). Cementitious materials for construction-scale 3D printing: Laboratory testing of fresh printing mixture. Constr. Build. Mater..

[27] Chen Y., Chaves Figueiredo S., Yalçinkaya Ç., Çopuroğlu O., Veer F., Schlangen E. (2019). The effect of viscosity-modifying admixture on the extrudability of limestone and calcined clay-based cementitious material for extrusion-based 3D concrete printing. Materials.

[28] Jayathilakage R., Rajeev P., Sanjayan J. (2020). Yield stress criteria to assess the buildability of 3D concrete printing. Constr. Build. Mater..

[29] Muthukrishnan S., Ramakrishnan S., Sanjayan J. (2021). Technologies for improving buildability in 3D concrete printing. Cem. Concr. Compos..

[30] Panda B., Ruan S., Unluer C., Tan M.J. (2019). Improving the 3D printability of high volume fly ash mixtures via the use of nano attapulgite clay. Compos. Part B Eng..

[31] Wu Y., Liu C., Liu H., Zhang Z., He C., Liu S., Zhang R., Wang Y., Bai G. (2021). Study on the rheology and buildability of 3D printed concrete with recycled coarse aggregates. J. Build. Eng..

[32] Zhang Y., Zhang Y., Liu G., Yang Y., Wu M., Pang B. (2018). Fresh properties of a novel 3D printing concrete ink. Constr. Build. Mater..

[33] Zhu B., Pan J., Nematollahi B., Zhou Z., Zhang Y., Sanjayan J. (2019). Development of 3D printable engineered cementitious composites with ultra-high tensile ductility for digital construction. Mater. Des..

[34] Bong S.H., Xia M., Nematollahi B., Shi C. (2021). Ambient temperature cured ‘just-add-water’geopolymer for 3D concrete printing applications. Cem. Concr. Compos..

[35] Nematollahi B., Vijay P., Sanjayan J., Nazari A., Xia M., Naidu Nerella V., Mechtcherine V. (2018). Effect of polypropylene fibre addition on properties of geopolymers made by 3D printing for digital construction. Materials.

[36] Tay Y.W.D., Panda B., Paul S.C., Noor Mohamed N.A., Tan M.J., Leong K.F. (2017). 3D printing trends in building and construction industry: A review. Virtual Phys. Prototyp..

[37] Ding T., Xiao J., Qin F., Duan Z. (2020). Mechanical behavior of 3D printed mortar with recycled sand at early ages. Constr. Build. Mater..

[38] Panda B., Lim J.H., Tan M.J. (2019). Mechanical properties and deformation behaviour of early age concrete in the context of digital construction. Compos. Part B Eng..

[39] Zou S., Xiao J., Ding T., Duan Z., Zhang Q. (2021). Printability and advantages of 3D printing mortar with 100% recycled sand. Constr. Build. Mater..

[40] Quanji Z., Lomboy G.R., Wang K. (2014). Influence of nano-sized highly purified magnesium alumino silicate clay on thixotropic behavior of fresh cement pastes. Constr. Build. Mater..

[41] Yuan Q., Zhou D., Li B., Huang H., Shi C. (2018). Effect of mineral admixtures on the structural build-up of cement paste. Constr. Build. Mater..

[42] Rehman A.U., Kim J.H. (2021). 3D concrete printing: A systematic review of rheology, mix designs, mechanical, microstructural, and durability characteristics. Materials.

[43] Perrot A., Rangeard D., Pierre A. (2016). Structural built-up of cement-based materials used for 3D-printing extrusion techniques. Mater. Struct..

[44] Rahul A., Santhanam M., Meena H., Ghani Z. (2019). 3D printable concrete: Mixture design and test methods. Cem. Concr. Compos..

[45] Kruger J., Zeranka S., van Zijl G. (2019). An ab initio approach for thixotropy characterisation of (nanoparticle-infused) 3D printable concrete. Constr. Build. Mater..

[46] Kruger J., Cho S., Zeranka S., Viljoen C., van Zijl G. (2020). 3D concrete printer parameter optimisation for high rate digital construction avoiding plastic collapse. Compos. Part B Eng..

[47] Papachristoforou M., Mitsopoulos V., Stefanidou M. (2018). Evaluation of workability parameters in 3D printing concrete. Procedia Struct. Integr..

[48] Weng Y., Li M., Tan M.J., Qian S. (2018). Design 3D printing cementitious materials via Fuller Thompson theory and Marson-Percy model. Constr. Build. Mater..

[49] Panda B., Noor Mohamed N.A., Paul S.C., Bhagath Singh G., Tan M.J., Šavija B. (2019). The Effect of Material Fresh Properties and Process Parameters on Buildability and Interlayer Adhesion of 3D Printed Concrete. Materials.

[50] Moeini M.A., Hosseinpoor M., Yahia A. (2020). Effectiveness of the rheometric methods to evaluate the build-up of cementitious mortars used for 3D printing. Constr. Build. Mater..

[51] Panda B., Tan M.J. (2018). Experimental study on mix proportion and fresh properties of fly ash based geopolymer for 3D concrete printing. Ceram. Int..

[52] Chen M., Yang L., Zheng Y., Huang Y., Li L., Zhao P., Wang S., Lu L., Cheng X. (2020). Yield stress and thixotropy control of 3D-printed calcium sulfoaluminate cement composites with metakaolin related to structural build-up. Constr. Build. Mater..

[53] Zhang Y., Zhang Y., She W., Yang L., Liu G., Yang Y. (2019). Rheological and harden properties of the high-thixotropy 3D printing concrete. Constr. Build. Mater..

[54] Jayathilakage R., Sanjayan J., Rajeev P. (2021). Comparison of rheology measurement techniques used in 3D concrete printing applications. Lecture Notes in Civil Engineering, Proceedings of the 10th International Conference on Structural Engineering and Construction Management, (ICSECM 2019), Chicago, IL USA, 20–25 May 2019.

[55] Nair S.A., Panda S., Santhanam M., Sant G., Neithalath N. (2020). A critical examination of the influence of material characteristics and extruder geometry on 3D printing of cementitious binders. Cem. Concr. Compos..

[56] Chen M., Li L., Wang J., Huang Y., Wang S., Zhao P., Lu L., Cheng X. (2020). Rheological parameters and building time of 3D printing sulphoaluminate cement paste modified by retarder and diatomite. Constr. Build. Mater..

[57] Chen M., Liu B., Li L., Cao L., Huang Y., Wang S., Zhao P., Lu L., Cheng X. (2020). Rheological parameters, thixotropy and creep of 3D-printed calcium sulfoaluminate cement composites modified by bentonite. Compos. Part B Eng..

[58] Ouyang J., Tan Y., Corr D.J., Shah S.P. (2016). The thixotropic behavior of fresh cement asphalt emulsion paste. Constr. Build. Mater..

[59] Qian Y., Lesage K., El Cheikh K., De Schutter G. (2018). Effect of polycarboxylate ether superplasticizer (PCE) on dynamic yield stress, thixotropy and flocculation state of fresh cement pastes in consideration of the Critical Micelle Concentration (CMC). Cem. Concr. Res..

[60] Jayathilakage R., Rajeev P., Sanjayan J. (2022). Rheometry for Concrete 3D Printing: A Review and an Experimental Comparison. Buildings.

[61] Lu G., Wang K. (2010). Investigation into Yield Behavior of Fresh Cement Paste: Model and Experiment. ACI Mater. J..

[62] Yalçınkaya Ç. (2022). Influence of Hydroxypropyl Methylcellulose Dosage on the Mechanical Properties of 3D Printable Mortars with and without Fiber Reinforcement. Buildings.

[63] Ma G., Li Z., Wang L., Wang F., Sanjayan J. (2019). Mechanical anisotropy of aligned fiber reinforced composite for extrusion-based 3D printing. Constr. Build. Mater..

[64] Wolfs R., Bos F., Salet T. (2019). Hardened properties of 3D printed concrete: The influence of process parameters on interlayer adhesion. Cem. Concr. Res..

[65] Ding T., Xiao J., Zou S., Zhou X. (2020). Anisotropic behavior in bending of 3D printed concrete reinforced with fibers. Compos. Struct..

[66] Mohammed A., Al-Saadi N.T.K. (2020). Ultra-high early strength cementitious grout suitable for additive manufacturing applications fabricated by using graphene oxide and viscosity modifying agents. Polymers.

[67] Ji G., Xiao J., Zhi P., Wu Y.C., Han N. (2022). Effects of extrusion parameters on properties of 3D printing concrete with coarse aggregates. Constr. Build. Mater..

[68] Liu H., Liu C., Bai G., Wu Y., He C., Zhang R., Wang Y. (2022). Influence of pore defects on the hardened properties of 3D printed concrete with coarse aggregate. Addit. Manuf..

[69] Liu H., Liu C., Wu Y., Bai G., He C., Zhang R., Wang Y. (2022). Hardened properties of 3D printed concrete with recycled coarse aggregate. Cem. Concr. Res..

[70] Rahul A., Mohan M.K., De Schutter G., Van Tittelboom K. (2022). 3D printable concrete with natural and recycled coarse aggregates: Rheological, mechanical and shrinkage behaviour. Cem. Concr. Compos..

[71] Wang X., Jia L., Jia Z., Zhang C., Chen Y., Ma L., Wang Z., Deng Z., Banthia N., Zhang Y. (2022). Optimization of 3D printing concrete with coarse aggregate via proper mix design and printing process. J. Build. Eng..

[72] Skibicki S., Kaszyńska M., Wahib N., Techman M., Federowicz K., Zieliński A., Wróblewski T., Olczyk N., Hoffmann M. (2020). Properties of composite modified with limestone powder for 3D concrete printing. Proceedings of the Second RILEM International Conference on Concrete and Digital Fabrication: Digital Concrete 2020.

[73] Ma G., Wang L., Ju Y. (2018). State-of-the-art of 3D printing technology of cementitious material—An emerging technique for construction. Sci. China Technol. Sci..

[74] Zhu W., Bartos P.J., Porro A. (2004). Application of nanotechnology in construction: Summary of a State-of-the-art Report. Mater. Struct..

[75] Pacheco-Torgal F., Jalali S. (2011). Nanotechnology: Advantages and drawbacks in the field of construction and building materials. Constr. Build. Mater..

[76] Mobasser S., Firoozi A.A. (2016). Review of nanotechnology applications in science and engineering. J. Civil Eng. Urban.

[77] Wu Z., Shi C., Khayat K.H., Wan S. (2016). Effects of different nanomaterials on hardening and performance of ultra-high strength concrete (UHSC). Cem. Concr. Compos..

[78] Rana A.K., Rana S.B., Kumari A., Kiran V. (2009). Significance of nanotechnology in construction engineering. Int. J. Recent Trends Eng..

[79] Dahlan A.S. (2021). Impact of nanotechnology on high performance cement and concrete. J. Mol. Struct..

[80] Sanchez F., Sobolev K. (2010). Nanotechnology in concrete—A review. Constr. Build. Mater..

[81] Sobolev K. Nanotechnology and nanoengineering of construction materials. Proceedings of the Nanotechnology in Construction (NICOM5).

[82] Silvestre J., Silvestre N., De Brito J. (2016). Review on concrete nanotechnology. Eur. J. Environ. Civ. Eng..

[83] Xie J., Zhang H., Duan L., Yang Y., Yan J., Shan D., Liu X., Pang J., Chen Y., Li X. (2020). Effect of nano metakaolin on compressive strength of recycled concrete. Constr. Build. Mater..

[84] Wang W.C. (2017). Compressive strength and thermal conductivity of concrete with nanoclay under Various High-Temperatures. Constr. Build. Mater..

[85] Teng L., Zhu J., Khayat K.H., Liu J. (2020). Effect of welan gum and nanoclay on thixotropy of UHPC. Cem. Concr. Res..

[86] Niu X.J., Li Q.B., Hu Y., Tan Y.S., Liu C.F. (2021). Properties of cement-based materials incorporating nano-clay and calcined nano-clay: A review. Constr. Build. Mater..

[87] Mehrabi P., Shariati M., Kabirifar K., Jarrah M., Rasekh H., Trung N.T., Shariati A., Jahandari S. (2021). Effect of pumice powder and nano-clay on the strength and permeability of fiber-reinforced pervious concrete incorporating recycled concrete aggregate. Constr. Build. Mater..

[88] Lei Z., Li Z., Zhang X., Shi X. (2021). Durability of CFRP-wrapped concrete in cold regions: A laboratory evaluation of montmorillonite nanoclay-modified siloxane epoxy adhesive. Constr. Build. Mater..

[89] Langaroudi M.A.M., Mohammadi Y. (2018). Effect of nano-clay on workability, mechanical, and durability properties of self-consolidating concrete containing mineral admixtures. Constr. Build. Mater..

[90] Irshidat M.R., Al-Saleh M.H. (2018). Thermal performance and fire resistance of nanoclay modified cementitious materials. Constr. Build. Mater..

[91] Heikal M., Abdel-Gawwad H.A., Ababneh F.A. (2018). Positive impact performance of hybrid effect of nano-clay and silica nano-particles on composite cements. Constr. Build. Mater..

[92] Hamed N., El-Feky M., Kohail M., Nasr E.S.A. (2019). Effect of nano-clay de-agglomeration on mechanical properties of concrete. Constr. Build. Mater..

[93] Yue Y., Zhou Y., Xing F., Gong G., Hu B., Guo M. (2020). An industrial applicable method to improve the properties of recycled aggregate concrete by incorporating nano-silica and micro-CaCO_3_. J. Clean. Prod..

[94] Yu R., Spiesz P., Brouwers H. (2014). Effect of nano-silica on the hydration and microstructure development of Ultra-High Performance Concrete (UHPC) with a low binder amount. Constr. Build. Mater..

[95] Wang J., Cheng Y., Yuan L., Xu D., Du P., Hou P., Zhou Z., Cheng X., Liu S., Wang Y. (2020). Effect of nano-silica on chemical and volume shrinkage of cement-based composites. Constr. Build. Mater..

[96] Sikora P., Rucinska T., Stephan D., Chung S.Y., Abd Elrahman M. (2020). Evaluating the effects of nanosilica on the material properties of lightweight and ultra-lightweight concrete using image-based approaches. Constr. Build. Mater..

[97] Raheem A., Abdulwahab R., Kareem M. (2021). Incorporation of metakaolin and nanosilica in blended cement mortar and concrete—A review. J. Clean. Prod..

[98] Liu Y., Chen B., Qin Z. (2020). Effect of nano-silica on properties and microstructures of magnesium phosphate cement. Constr. Build. Mater..

[99] Horszczaruk E., Sikora P., Cendrowski K., Mijowska E. (2017). The effect of elevated temperature on the properties of cement mortars containing nanosilica and heavyweight aggregates. Constr. Build. Mater..

[100] Gunasekara C., Sandanayake M., Zhou Z., Law D.W., Setunge S. (2020). Effect of nano-silica addition into high volume fly ash–hydrated lime blended concrete. Constr. Build. Mater..

[101] Abhilash P., Nayak D.K., Sangoju B., Kumar R., Kumar V. (2021). Effect of nano-silica in concrete; a review. Constr. Build. Mater..

[102] Reches Y., Thomson K., Helbing M., Kosson D.S., Sanchez F. (2018). Agglomeration and reactivity of nanoparticles of SiO_2_, TiO_2_, Al_2_O_3_, Fe_2_O_3_, and clays in cement pastes and effects on compressive strength at ambient and elevated temperatures. Constr. Build. Mater..

[103] Oltulu M., Şahin R. (2013). Effect of nano-SiO_2_, nano-Al_2_O_3_ and nano-Fe_2_O_3_ powders on compressive strengths and capillary water absorption of cement mortar containing fly ash: A comparative study. Energy Build..

[104] Ng D.S., Paul S.C., Anggraini V., Kong S.Y., Qureshi T.S., Rodriguez C.R., Liu Q.F., Šavija B. (2020). Influence of SiO_2_, TiO_2_ and Fe_2_O_3_ nanoparticles on the properties of fly ash blended cement mortars. Constr. Build. Mater..

[105] Madandoust R., Mohseni E., Mousavi S.Y., Namnevis M. (2015). RETRACTED: An experimental investigation on the durability of self-compacting mortar containing nano-SiO_2_, nano-Fe_2_O_3_ and nano-CuO, 2015. Constr. Build. Mater..

[106] Kani E.N., Rafiean A.H., Alishah A., Astani S.H., Ghaffar S.H. (2021). The effects of Nano-Fe_2_O_3_ on the mechanical, physical and microstructure of cementitious composites. Constr. Build. Mater..

[107] Joshaghani A., Balapour M., Mashhadian M., Ozbakkaloglu T. (2020). Effects of nano-TiO_2_, nano-Al_2_O_3_, and nano-Fe_2_O_3_ on rheology, mechanical and durability properties of self-consolidating concrete (SCC): An experimental study. Constr. Build. Mater..

[108] Heikal M., Zaki M.E., Ibrahim S.M. (2021). Characterization, hydration, durability of nano-Fe_2_O_3_-composite cements subjected to sulphates and chlorides media. Constr. Build. Mater..

[109] Feng H., Zhao X., Li L., Zhao X., Gao D. (2021). Water stability of bonding properties between nano-Fe_2_O_3_-modified magnesium-phosphate-cement mortar and steel fibre. Constr. Build. Mater..

[110] Feng H., Wang Z., Sheikh M.N., Zhao X., Gao D., Hadi M.N. (2019). The effect of nano-SiO_2_, nano-Al_2_O_3_, and nano-Fe_2_O_3_ on the compressive strength and workability of magnesium phosphate cement-based mortar. Adv. Civ. Eng. Mater..

[111] Cibilakshmi G., Jegan J. (2020). A DOE approach to optimize the strength properties of concrete incorporated with different ratios of PVA fibre and nano-Fe_2_O_3_. Adv. Compos. Lett..

[112] Daniyal M., Akhtar S., Azam A. (2019). Effect of nano-TiO_2_ on the properties of cementitious composites under different exposure environments. J. Mater. Res. Technol..

[113] Meng T., Yu Y., Qian X., Zhan S., Qian K. (2012). Effect of nano-TiO_2_ on the mechanical properties of cement mortar. Constr. Build. Mater..

[114] Sun J., Tian L., Yu Z., Zhang Y., Li C., Hou G., Shen X. (2020). Studies on the size effects of nano-TiO_2_ on Portland cement hydration with different water to solid ratios. Constr. Build. Mater..

[115] Yang L., Jia Z., Zhang Y., Dai J. (2015). Effects of nano-TiO_2_ on strength, shrinkage and microstructure of alkali activated slag pastes. Cem. Concr. Compos..

[116] Zhang R., Cheng X., Hou P., Ye Z. (2015). Influences of nano-TiO2 on the properties of cement-based materials: Hydration and drying shrinkage. Constr. Build. Mater..

[117] Gao Y., Jing H., Yu Z., Li L., Wu J., Chen W. (2022). Particle size distribution of aggregate effects on the reinforcing roles of carbon nanotubes in enhancing concrete ITZ. Constr. Build. Mater..

[118] Madhavi T.C., Pavithra P., Singh S.B., Raj S.V., Paul S. (2013). Effect of multiwalled carbon nanotubes on mechanical properties of concrete. Int. J. Sci. Res..

[119] Mohsen M.O., Al Ansari M.S., Taha R., Al Nuaimi N., Taqa A.A. (2019). Carbon nanotube effect on the ductility, flexural strength, and permeability of concrete. J. Nanomater..

[120] Siddique R., Mehta A. (2014). Effect of carbon nanotubes on properties of cement mortars. Constr. Build. Mater..

[121] Yao Y., Lu H. (2021). Mechanical properties and failure mechanism of carbon nanotube concrete at high temperatures. Constr. Build. Mater..

[122] Camiletti J., Soliman A.M., Nehdi M.L. (2013). Effect of nano-calcium carbonate on early-age properties of ultra-high-performance concrete. Mag. Concr. Res..

[123] Feng J., Yang F., Qian S. (2021). Improving the bond between polypropylene fiber and cement matrix by nano calcium carbonate modification. Constr. Build. Mater..

[124] Luan C., Zhou Y., Liu Y., Ren Z., Wang J., Yuan L., Du S., Zhou Z., Huang Y. (2022). Effects of nano-SiO_2_, nano-CaCO_3_ and nano-TiO_2_ on properties and microstructure of the high content calcium silicate phase cement (HCSC). Constr. Build. Mater..

[125] Ren Z., Liu Y., Yuan L., Luan C., Wang J., Cheng X., Zhou Z. (2021). Optimizing the content of nano-SiO_2_, nano-TiO_2_ and nano-CaCO_3_ in Portland cement paste by response surface methodology. J. Build. Eng..

[126] Shaikh F.U., Supit S.W. (2014). Mechanical and durability properties of high volume fly ash (HVFA) concrete containing calcium carbonate (CaCO_3_) nanoparticles. Constr. Build. Mater..

[127] Li J., Cheng G., Huang S., Lian P. (2021). Effect of ZnO on the whiteness of white Portland cement clinker. Cem. Concr. Res..

[128] Li X., Li J., Lu Z., Chen J. (2021). Properties and hydration mechanism of cement pastes in presence of nano-ZnO. Constr. Build. Mater..

[129] Liu J., Jin H., Gu C., Yang Y. (2019). Effects of zinc oxide nanoparticles on early-age hydration and the mechanical properties of cement paste. Constr. Build. Mater..

[130] Barbhuiya S., Mukherjee S., Nikraz H. (2014). Effects of nano-Al_2_O_3_ on early-age microstructural properties of cement paste. Constr. Build. Mater..

[131] Feng H., Shen S., Pang Y., Gao D., Wang Z., Sheikh M.N. (2021). Mechanical properties of fiber and nano-Al_2_O_3_ reinforced magnesium phosphate cement composite. Constr. Build. Mater..

[132] Liu X., Ma B., Tan H., Li H., Mei J., Zhang T., Chen P., Gu B. (2019). Chloride immobilization of cement-based material containing nano-Al_2_O_3_. Constr. Build. Mater..

[133] Yang Z., Gao Y., Mu S., Chang H., Sun W., Jiang J. (2019). Improving the chloride binding capacity of cement paste by adding nano-Al_2_O_3_. Constr. Build. Mater..

[134] Yang Z., Sui S., Wang L., Feng T., Gao Y., Mu S., Tang L., Jiang J. (2020). Improving the chloride binding capacity of cement paste by adding nano-Al2O3: The cases of blended cement pastes. Constr. Build. Mater..

[135] Zhang A., Yang W., Ge Y., Du Y., Liu P. (2021). Effects of nano-SiO2 and nano-Al2O3 on mechanical and durability properties of cement-based materials: A comparative study. J. Build. Eng..

[136] Chu H., Qin J., Gao L., Jiang J., Wang F., Wang D. (2022). Effects of graphene oxide on mechanical properties and microstructure of ultra-high-performance lightweight concrete. J. Sustain.-Cem.-Based Mater..

[137] Devi S., Khan R. (2020). Effect of graphene oxide on mechanical and durability performance of concrete. J. Build. Eng..

[138] Jamnam S., Maho B., Techaphatthanakon A., Ruttanapun C., Aemlaor P., Zhang H., Sukontasukkul P. (2022). Effect of graphene oxide nanoparticles on blast load resistance of steel fiber reinforced concrete. Constr. Build. Mater..

[139] Liu C., Hunag X., Wu Y.Y., Deng X., Zheng Z., Yang B. (2022). Studies on mechanical properties and durability of steel fiber reinforced concrete incorporating graphene oxide. Cem. Concr. Compos..

[140] Maglad A.M., Zaid O., Arbili M.M., Ascensão G., Șerbănoiu A.A., Grădinaru C.M., García R.M., Qaidi S.M., Althoey F., de Prado-Gil J. (2022). A study on the properties of geopolymer concrete modified with nano graphene oxide. Buildings.

[141] Sui Y., Liu S., Ou C., Liu Q., Meng G. (2021). Experimental investigation for the influence of graphene oxide on properties of the cement-waste concrete powder composite. Constr. Build. Mater..

[142] Tonelli M., Gelli R., Ridi F., Baglioni P. (2021). Magnesium phosphate-based cements containing Halloysite nanotubes for cracks repair. Constr. Build. Mater..

[143] Tonelli M., Baglioni P., Ridi F. (2020). Halloysite nanotubes as nano-carriers of corrosion inhibitors in cement formulations. Materials.

[144] Haw T.T., Hart F., Rashidi A., Pasbakhsh P. (2020). Sustainable cementitious composites reinforced with metakaolin and halloysite nanotubes for construction and building applications. Appl. Clay Sci..

[145] Ghoddousi P., Zareechian M., Javid A.A.S., Korayem A.H. (2020). Microstructural study and surface properties of concrete pavements containing nanoparticles. Constr. Build. Mater..

[146] Farzadnia N., Ali A.A.A., Demirboga R., Anwar M.P. (2013). Effect of halloysite nanoclay on mechanical properties, thermal behavior and microstructure of cement mortars. Cem. Concr. Res..

[147] Farzadnia N. (2019). Mechanical properties and chemical characterization of mortars with halloysite nanoclay after exposure to elevated temperatures. Adv. Civ. Eng. Mater..

[148] Anjum G., Kumar N. (2018). Investigation on influence of halloysite nanoclay and carbon nanotubes on cement nano composites-A critical review. Int. J. Mater. Eng..

[149] Allalou S., Kheribet R., Benmounah A. (2019). Effects of calcined halloysite nano-clay on the mechanical properties and microstructure of low-clinker cement mortar. Case Stud. Constr. Mater..

[150] Meddah M., Praveenkumar T., Vijayalakshmi M., Manigandan S., Arunachalam R. (2020). Mechanical and microstructural characterization of rice husk ash and Al2O3 nanoparticles modified cement concrete. Constr. Build. Mater..

[151] Sujay H., Nair N.A., Rao H.S., Sairam V. (2020). Experimental study on durability characteristics of composite fiber reinforced high-performance concrete incorporating nanosilica and ultra fine fly ash. Constr. Build. Mater..

[152] Sheikh T.M., Anwar M.P., Muthoosamy K., Jaganathan J., Chan A., Mohamed A.A. (2021). The mechanics of carbon-based nanomaterials as cement reinforcement: A critical review. Constr. Build. Mater..

[153] Ralegaonkar R., Gavali H., Aswath P., Abolmaali S. (2018). Application of chopped basalt fibers in reinforced mortar: A review. Constr. Build. Mater..

[154] Aly T., Sanjayan J.G., Collins F. (2008). Effect of polypropylene fibers on shrinkage and cracking of concretes. Mater. Struct..

[155] Han J., Zhao M., Chen J., Lan X. (2019). Effects of steel fiber length and coarse aggregate maximum size on mechanical properties of steel fiber reinforced concrete. Constr. Build. Mater..

[156] Liu B., Guo J., Zhou J., Wen X., Deng Z., Wang H., Zhang X. (2020). The mechanical properties and microstructure of carbon fibers reinforced coral concrete. Constr. Build. Mater..

[157] Deshmukh S., Bhusari J., Zende A. (2012). Effect of glass fibers on ordinary Portland cement concrete. IOSR J. Eng..

[158] Yu H., Meng T., Zhao Y., Liao J., Ying K. (2022). Effects of basalt fiber powder on mechanical properties and microstructure of concrete. Case Stud. Constr. Mater..

[159] Machaka M.M., Basha H.S., ElKordi A.M. (2014). The effect of using fan palm natural fibers on the mechanical properties and durability of concrete. Int. J. Mater. Sci. Eng.

[160] Moelich G.M., Kruger J., Combrinck R. (2020). Plastic shrinkage cracking in 3D printed concrete. Compos. Part B Eng..

[161] Federowicz K., Kaszyńska M., Zieliński A., Hoffmann M. (2020). Effect of curing methods on shrinkage development in 3D-printed concrete. Materials.

[162] Cohen M.D., Olek J., Dolch W.L. (1990). Mechanism of plastic shrinkage cracking in portland cement and portland cement-silica fume paste and mortar. Cem. Concr. Res..

[163] De Schutter G., Lesage K., Mechtcherine V., Nerella V.N., Habert G., Agusti-Juan I. (2018). Vision of 3D printing with concrete—Technical, economic and environmental potentials. Cem. Concr. Res..

[164] Shakor P., Nejadi S., Paul G. (2019). A Study into the Effect of Different Nozzles Shapes and Fibre-Reinforcement in 3D Printed Mortar. Materials.

[165] Panda B., Unluer C., Tan M.J. (2018). Investigation of the rheology and strength of geopolymer mixtures for extrusion-based 3D printing. Cem. Concr. Compos..

[166] Panda B., Paul S.C., Mohamed N.A.N., Tay Y.W.D., Tan M.J. (2018). Measurement of tensile bond strength of 3D printed geopolymer mortar. Measurement.

[167] Lu B., Zhu W., Weng Y., Liu Z., Yang E.H., Leong K.F., Tan M.J., Wong T.N., Qian S. (2020). Study of MgO-activated slag as a cementless material for sustainable spray-based 3D printing. J. Clean. Prod..

[168] Beersaerts G., Lucas S.S., Pontikes Y. (2020). An Fe-Rich Slag-Based Mortar for 3D Printing. Proceedings of the Second RILEM International Conference on Concrete and Digital Fabrication: Digital Concrete.

[169] Al-Qutaifi S., Nazari A., Bagheri A. (2018). Mechanical properties of layered geopolymer structures applicable in concrete 3D-printing. Constr. Build. Mater..

[170] Mazhoud B., Perrot A., Picandet V., Rangeard D., Courteille E. (2019). Underwater 3D printing of cement-based mortar. Constr. Build. Mater..

[171] Cho S., Kruger J., van Rooyen A., Zeranka S., van Zijl G., Mechtcherine V., Khayat K., Secrieru E. (2020). Rheology of 3D Printable Lightweight Foam Concrete Incorporating Nano-Silica. Proceedings of the Rheology and Processing of Construction Materials.

[172] Markin V., Krause M., Otto J., Schröfl C., Mechtcherine V. (2021). 3D-printing with foam concrete: From material design and testing to application and sustainability. J. Build. Eng..

[173] Liu C., Wang X., Chen Y., Zhang C., Ma L., Deng Z., Chen C., Zhang Y., Pan J., Banthia N. (2021). Influence of hydroxypropyl methylcellulose and silica fume on stability, rheological properties, and printability of 3D printing foam concrete. Cem. Concr. Compos..

[174] Furet B., Poullain P., Garnier S. (2019). 3D printing for construction based on a complex wall of polymer-foam and concrete. Addit. Manuf..

[175] Falliano D., De Domenico D., Ricciardi G., Gugliandolo E. (2020). 3D-printable lightweight foamed concrete and comparison with classical foamed concrete in terms of fresh state properties and mechanical strength. Constr. Build. Mater..

[176] Falliano D., Crupi G., De Domenico D., Ricciardi G., Restuccia L., Ferro G., Gugliandolo E., Bos F.P., Lucas S.S., Wolfs R.J., Salet T.A. (2020). Investigation on the Rheological Behavior of Lightweight Foamed Concrete for 3D Printing Applications. Proceedings of the Second RILEM International Conference on Concrete and Digital Fabrication.

[177] Cho S., Kruger J., Zeranka S., van Rooyen A., van Zijl G. Mechanical evaluation of 3D printable nano-silica incorporated fibre-reinforced lightweight foam concrete. Proceedings of the 10th International Conference on Fracture Mechanics of Concrete and Concrete Structures.

[178] Cho S., Kruger J., van Rooyen A., van Zijl G. (2021). Rheology and application of buoyant foam concrete for digital fabrication. Compos. Part B Eng..

[179] Zhu B., Pan J., Zhou Z., Cai J. (2021). Mechanical properties of engineered cementitious composites beams fabricated by extrusion-based 3D printing. Eng. Struct..

[180] Zhang Y., Aslani F. (2021). Development of fibre reinforced engineered cementitious composite using polyvinyl alcohol fibre and activated carbon powder for 3D concrete printing. Constr. Build. Mater..

[181] Yu K., McGee W., Ng T.Y., Zhu H., Li V.C. (2021). 3D-printable engineered cementitious composites (3DP-ECC): Fresh and hardened properties. Cem. Concr. Res..

[182] Chaves Figueiredo S., Romero Rodríguez C., Ahmed Z.Y., Bos D., Xu Y., Salet T.M., Çopuroğlu O., Schlangen E., Bos F.P. (2019). An approach to develop printable strain hardening cementitious composites. Mater. Des..

[183] Zhao Z., Chen M., Zhong X., Huang Y., Yang L., Zhao P., Wang S., Lu L., Cheng X. (2021). Effects of bentonite, diatomite and metakaolin on the rheological behavior of 3D printed magnesium potassium phosphate cement composites. Addit. Manuf..

[184] Zhao Z., Chen M., Xu J., Li L., Huang Y., Yang L., Zhao P., Lu L. (2021). Mix design and rheological properties of magnesium potassium phosphate cement composites based on the 3D printing extrusion system. Constr. Build. Mater..

[185] Weng Y., Ruan S., Li M., Mo L., Unluer C., Tan M.J., Qian S. (2019). Feasibility study on sustainable magnesium potassium phosphate cement paste for 3D printing. Constr. Build. Mater..

[186] Khalil A., Wang X., Celik K. (2020). 3D printable magnesium oxide concrete: Towards sustainable modern architecture. Addit. Manuf..

[187] Cao X., Li Z., Sanjayan J.G., Nazari A., Nematollahi B. (2019). Factors Influencing the Mechanical Properties of Three-Dimensional Printed Products From Magnesium Potassium Phosphate Cement Material. 3D Concrete Printing Technology.

[188] Tarhan Y., Tarhan I., Craveiro F., Bartolo H. (2021). Sustainable Materials for Additive Manufacturing: Earth-Based Concrete. Proceedings of the International Conference on Water Energy Food and Sustainability.

[189] Perrot A., Rangeard D., Courteille E. (2018). 3D printing of earth-based materials: Processing aspects. Constr. Build. Mater..

[190] Gomaa M., Jabi W., Veliz Reyes A., Soebarto V. (2021). 3D printing system for earth-based construction: Case study of cob. Autom. Constr..

[191] Tobon J.I., Reales O.M., Restrepo O.J., Borrachero M.V., Paya J. (2018). Effect of Pyrogenic Silica and Nanosilica on Portland Cement Matrices. J. Mater. Civ. Eng..

[192] Mendoza Reales O.A., Duda P., Silva E.C., Paiva M.D., Filho R.D.T. (2019). Nanosilica particles as structural buildup agents for 3D printing with Portland cement pastes. Constr. Build. Mater..

[193] Kwan A., Li Y. (2013). Effects of fly ash microsphere on rheology, adhesiveness and strength of mortar. Constr. Build. Mater..

[194] Kruger J., Van den Heever M., Cho S., Zeranka S., Van Zijl G.P.A.G. High-performance 3D printable concrete enhanced with nanomaterials. Proceedings of the International Conference On Sustainable Materials, Systems and Structures (SMSS 2019).

[195] Jiang Q., Liu Q., Wu S., Zheng H., Sun W. (2022). Modification effect of nanosilica and polypropylene fiber for extrusion-based 3D printing concrete: Printability and mechanical anisotropy. Addit. Manuf..

[196] Srinivas D., Dey D., Panda B., Sitharam T.G. (2022). Printability, Thermal and Compressive Strength Properties of Cementitious Materials: A Comparative Study with Silica Fume and Limestone. Materials.

[197] Van den Heever M., Bester F.A., Kruger P.J., van Zijl G.P.A.G. Effect of Silicon Carbide (SiC) nanoparticles on 3D printability of cement-based materials. Proceedings of the Advances in Engineering Materials, Structures and Systems: Innovations, Mecahnics and Applications.

[198] Vickers N.J.N. (2017). Animal communication: When i’m calling you, will you answer too?. Curr. Biol..

[199] Siddique R., Klaus J. (2009). Influence of metakaolin on the properties of mortar and concrete: A review. Appl. Clay Sci..

[200] Qian Y., Kawashima S. (2016). Use of creep recovery protocol to measure static yield stress and structural rebuilding of fresh cement pastes. Cem. Concr. Res..

[201] Marchon D., Kawashima S., Bessaies-Bey H., Mantellato S., Ng S. (2018). Hydration and rheology control of concrete for digital fabrication: Potential admixtures and cement chemistry. Cem. Concr. Res..

[202] Kuder K., Shah S. (2007). Rheology of extruded cement-based materials. ACI Mater. J..

[203] Kim J., Beacraft M., Shah S. (2010). Effect of mineral admixtures on formwork pressure of self-consolidating concrete. Cem. Concr. Comp..

[204] Kawashima S., Chaouche M., Corr D., Shah S. (2013). Rate of thixotropic rebuilding of cement pastes modified with highly purified attapulgite clays. Cem. Concr. Res..

[205] Kaci A., Chaouche M., Andréani P. (2011). Influence of bentonite clay on the rheological behaviour of fresh mortars. Cem. Concr. Res..

[206] Ferron R., Shah S., Fuente E., Negro C. (2013). Aggregation and breakage kinetics of fresh cement paste. Cem. Concr. Res..

[207] Conte T., Chaouche M. (2016). Rheological behavior of cement pastes under large amplitude oscillatory shear. Cem. Concr. Res..

[208] Varela H., Barluenga G., Palomar I. (2020). Influence of nanoclays on flowability and rheology of SCC pastes. Constr. Build. Mater..

[209] Sayanthan R., Shravan M., Jay S., Kirubajiny P. (2021). Concrete 3D printing of lightweight elements using hollow-core extrusion of filaments. Cem. Concr. Compos..

[210] Qian Y., Ma S., Kawashima S., De Schutter G. (2019). Rheological characterization of the viscoelastic solid-like properties of fresh cement pastes with nanoclay addition. Theor. Appl. Fract. Mech..

[211] Qian Y., De Schutter G. (2018). Enhancing thixotropy of fresh cement pastes with nanoclay in presence of polycarboxylate ether superplasticizer (PCE). Cem. Concr. Res..

[212] Dai X., Ren Q., Aydın S. (2021). and Yardımcı, M.Y.; Lesage, K.; De Schutter, G. Enhancing thixotropy and structural build-up of alkali-activated slag/fly ash pastes with nano clay. Mater. Struct..

[213] Panda B., Unluer C., Tan M.J. (2019). Extrusion and rheology characterization of geopolymer nanocomposites used in 3D printing. Compos. Part B Eng..

[214] Panda B., Ruan S., Unluer C., Tan M.J. (2020). Investigation of the properties of alkali-activated slag mixes involving the use of nanoclay and nucleation seeds for 3D printing. Compos. Part B Eng..

[215] Ferron R.P., Gregori A., Sun Z., Shah S.P. (2007). Rheological method to evaluate structural buildup in self-consolidating concrete cement pastes. ACI Mater. J..

[216] Vance K., Kumar A., Sant G., Neithalath N. (2013). The rheological properties of ternary binders containing Portland cement, limestone, and metakaolin or fly ash. Cem. Concr. Res..

[217] Kawashima S., Kim J.H., Corr D.J., Shah S.P. (2012). Study of the mechanisms underlying the fresh-state response of cementitious materials modified with nanoclays. Constr. Build. Mater..

[218] Tregger N.A., Pakula M.E., Shah S.P. (2010). Influence of clays on the rheology of cement pastes. Cem. Concr. Res..

[219] Nehdi M., Rahman M.A. (2004). Estimating rheological properties of cement pastes using various rheological models for different test geometry, gap and surface friction. Cem. Concr. Res..

[220] Lomboy G.R., Wang X., Wang K. (2014). Rheological behavior and formwork pressure of SCC, SFSCC, and NC mixtures. Cem. Concr. Compos..

[221] Kawashima S., Chaouche M., Corr D.J., Shah S.P. (2014). Influence of purified attapulgite clays on the adhesive properties of cement pastes as measured by the tack test. Cem. Concr. Compos..

[222] Olphen H.V., Hsu P. (1964). An Introduction to Clay Colloid Chemistry. Soil Sci..

[223] Bouras R., Chaouche M., Kaci S. (2008). Influence of Viscosity-Modifying Admixtures on the Thixotropic Behaviour of Cement Pastes. Appl. Rheol..

[224] Bellour M., Knaebel A., Harden J.L., Lequeux F., Munch J.P. (2003). Aging processes and scale dependence in soft glassy colloidal suspensions. Phys. Rev. E.

[225] Douba A., Ma S., Kawashima S. (2022). Rheology of fresh cement pastes modified with nanoclay-coated cements. Cem. Concr. Compos..

[226] Aydin E.M., Kara B., Bundur Z.B., Ozyurt N., Bebek O., Ali Gulgun M. (2022). A comparative evaluation of sepiolite and nano-montmorillonite on the rheology of cementitious materials for 3D printing. Constr. Build. Mater..

[227] Kaushik S., Sonebi M., Amato G., Perrot A., Das U.K. (2022). Influence of nanoclay on the fresh and rheological behaviour of 3D printing mortar. Mater. Today Proc..

[228] Muthu M., Yang E.H., Unluer C. (2021). Effect of graphene oxide on the deterioration of cement pastes exposed to citric and sulfuric acids. Cem. Concr. Compos..

[229] Luo J., Zhou C., Li W., Chen S., Habibnejad Korayem A., Duan W. (2021). Using graphene oxide to improve physical property and control ASR expansion of cement mortar. Constr. Build. Mater..

[230] Dong W., Huang Y., Lehane B., Aslani F., Ma G. (2021). Mechanical and electrical properties of concrete incorporating an iron particle contained nano graphite by product. Constr. Build. Mater..

[231] Adamu M., Trabanpruek P., Jongvivatsakul P., Likitlersuang S., Iwanami M. (2021). Mechanical performance and optimization of high volume fly ash concrete containing plastic wastes and graphene nanoplatelets using response surface methodology. Constr. Build. Mater..

[232] Alkhateb H., Al-Ostaz A., Cheng A.H.D., Li X. (2013). Materials genome for graphene-cement nanocomposites. J. Nanomechanics Micromechanics.

[233] Jing Z., Guo X.Z., Pei G.H., Zhi H.Y., De Chang J. (2017). 3D printing strong and conductive geo-polymer nanocomposite structures modified by graphene oxide. Carbon.

[234] Cui K., Chang J., Feo L., Chow C.L., Lau D. (2022). Developments and Applications of Carbon Nanotube Reinforced Cement-Based Composites as Functional Building Materials. Front. Mater..

[235] Dulaj A., Suijs M.P.M., Salet T.A.M., Lucas S.S., Buswell R., Blanco A., Cavalaro S., Kinnell P. (2022). Incorporation and Characterization of Multi-walled Carbon Nanotube Concrete Composites for 3D Printing Applications. Proceedings of the Third RILEM International Conference on Concrete and Digital Fabrication.

[236] Kan D., Liu G., Cao S.C., Chen Z., Lyu Q. (2022). Mechanical Properties and Pore Structure of Multiwalled Carbon Nanotube-Reinforced Reactive Powder Concrete for Three-Dimensional Printing Manufactured by Material Extrusion. 3D Print. Addit. Manuf..

[237] De Matos P., Zat T., Corazza K., Fensterseifer E., Sakata R., Mohamad G., Rodríguez E. (2022). Effect of TiO_2_ Nanoparticles on the Fresh Performance of 3D-Printed Cementitious Materials. Materials.

[238] Liu Q., Jiang Q., Huang M., Xin J., Chen P. (2022). The fresh and hardened properties of 3D printing cement-base materials with self-cleaning nano-TiO_2_: An exploratory study. J. Clean. Prod..

[239] Yang H., Che Y., Shi M. (2021). Influences of calcium carbonate nanoparticles on the workability and strength of 3D printing cementitious materials containing limestone powder. J. Build. Eng..

[240] Yang H., Li W., Che Y. (2020). 3D Printing Cementitious Materials Containing Nano-CaCO_3_: Workability, Strength, and Microstructure. Front. Mater..

[241] Ghantous R.M., Valadez-Carranza Y., Reese S.R., Weiss W.J. (2022). Drying behavior of 3D printed cementitious pastes containing cellulose nanocrystals. Cement.

[242] Valadez-Carranza C.Y., Moradllo M.K., Weiss W. Examining the effect of Cellulose Nanocrystals (CNC) on 3-D Printed Cement Composites. Proceedings of the TRB 1st International Conference on 3D printing and Transportation.

[243] Dey D., Srinivas D., Boddepalli U., Panda B., Gandhi I.S.R., Sitharam T. (2022). 3D printability of ternary Portland cement mixes containing fly ash and limestone. Mater. Today Proc..

[244] Kim K.K., Yeon J., Lee H.J., Yeon K.S. (2019). Feasibility Study of SBR-Modified Cementitious Mixtures for Use as 3D Additive Construction Materials. Polymers.

[245] Kim K.K., Yeon J., Lee H.J., Yeon K.S. (2019). Strength Development Characteristics of SBR-Modified Cementitious Mixtures for 3-Demensional Concrete Printing. Sustainability.

